# Chains of Commerce: A Comprehensive Review of Animal Welfare Impacts in the International Wildlife Trade

**DOI:** 10.3390/ani15070971

**Published:** 2025-03-27

**Authors:** Helen Lambert, Angie Elwin, Délagnon Assou, Mark Auliya, Lauren A. Harrington, Alice C. Hughes, Aniruddha Mookerjee, Tom Moorhouse, Gohar A. Petrossian, Evan Sun, Clifford Warwick, Özgün Emre Can, Neil D’Cruze

**Affiliations:** 1 Animal Welfare Consultancy, Newton Abbot TQ12 3BW, UK; 2 World Animal Protection, 222 Greys Inn Road, London WC1X 8HB, UK; angieelwin@worldanimalprotection.org (A.E.); evansun@worldanimalprotection.org.cn (E.S.); neildcruze@worldanimalprotection.org (N.D.); 3 Laboratory of Ecology and Ecotoxicology (LaEE), University of Lomé, Lomé 01 BP 1515, Togo; patricedelagnon@gmail.com; 4 Zoological Research Museum Alexander Koenig of the Leibniz Institute for the Analysis of Biodiversity Change, 53113 Bonn, Germany; markauliya@bonn.org; 5 Wildlife Conservation Research Unit, Department of Biology, University of Oxford, Tubney House, Tubney OX13 5QL, UK; lauren.harrington@biology.ox.ac.uk; 6 School of Biological Sciences, University of Hong Kong, Hong Kong; achughes@xtbg.ac.cn; 7 Independent Researcher, 11/4 Baherakhar, Malajkhand, Balaghat 481116, India; jhampanm@gmail.com; 8 Oxford Wildlife Research, 64 Charles Street, Oxford OX4 3AS, UK; tompmoorhouse@protonmail.com; 9 John Jay College of Criminal Justice, 524 West 59th Street, New York, NY 10019, USA; gpetrossian@jjay.cuny.edu; 10 Emergent Disease Foundation, 71–75 Shelton Street, Covent Garden, London WC2H 9JQ, UK; cliffordwarwick@gmail.com; 11 Department of Biology, Ankara University, Dögol Street, Ankara 06100, Turkey; oecan@ankara.edu.tr

**Keywords:** animal sentience, animal welfare, demand redirection, behaviour change initiatives, wildlife trade

## Abstract

The commercial wildlife trade involves billions of animals each year, consumed for various purposes, including food, fashion, entertainment, traditional medicine, and pets. The experiences of the animals involved vary widely, with negative welfare states being commonplace. To highlight the broad scope of animal welfare impacts across the commercial wildlife trade, we present ten case studies featuring a range of species traded globally for different purposes: (1) Ball pythons captured and farmed to serve as pets; (2) Zebrafish captive bred to serve as pets; (3) African Grey Parrots taken from the wild for the pet industry; (4) Sharks de-finned for traditional medicine; (5) Pangolins hunted for traditional medicine; (6) Crickets farmed for food and feed; (7) Frogs wild-caught for the frog-leg trade; (8) Crocodilians killed for their skins; (9) Lions farmed and killed for tourism; and (10) Elephants held captive for tourism. The case studies demonstrate that wild animals commercially traded can suffer from negative welfare states ranging from chronic stress and depression to frustration and extreme hunger. The individuals involved range from hundreds to billions, and their suffering can last a lifetime. Given the welfare issues identified and the growing recognition and scientific evidence for animal sentience, we propose reducing and redirecting consumer demand for these consumptive wildlife practices that negatively impact animals.

## Simple Summary

The commercial wildlife trade involves billions of animals annually consumed for various purposes, including food, fashion, entertainment, traditional medicine, and pets. Species affected range from mammals such as lions to insects such as crickets. In this review, we present ten case studies to highlight the animal welfare impacts of the commercial wildlife trade: (1) Ball pythons captured and farmed to serve as pets; (2) Zebrafish captive bred to serve as pets; (3) African Grey Parrots taken from the wild for the pet industry; (4) Sharks de-finned for traditional medicine; (5) Pangolins hunted for traditional medicine; (6) Crickets farmed for food and feed; (7) Frogs wild-caught for the frog-leg trade; (8) Crocodilians killed for their skins; (9) Lions farmed and killed for tourism; and (10) Elephants held captive for tourist rides. These case studies demonstrate that the average welfare experience of a wild animal being traded is negative and that most animals routinely experience negative states such as extreme hunger and thirst, pain, fear, and chronic stress. Therefore, we posit a new approach that seeks to mitigate these negative impacts by reducing and redirecting consumer demand away from the consumptive use of wildlife practices and towards sustainable, non-consumptive “wildlife friendly” alternatives.

## 1. Introduction

Wild animals are traded across multiple industries, including the pet trade, food, traditional medicine, and entertainment, driven by urban wealth, cultural practices, and commercial markets [1,2,3,4,5]. The global commercial trade in wild animals, distinct from local subsistence use, represents a vast, profit-driven industry involving billions of individuals annually, with complex supply chains and far-reaching impacts [1,2]. Accurate numbers of wild animals involved in trade are lacking because most commercial wildlife trade is unmonitored or performed illegally [6,7,8,9,10,11]. However, recent estimates suggest that there are over 7600 terrestrial vertebrate species and several thousand terrestrial invertebrate species known to be legally traded [6,7,8]. Furthermore, CITES records report that over the decade between 2005 and 2014, nearly 54 million CITES-listed individual wild vertebrates and 36.5 million invertebrates were exported worldwide [9]. These numbers only include the CITES-listed species, and many more non-CITES-listed species are known to be traded in high numbers [10].

The commercial wildlife trade affects animals in different ways, with the extent and duration of suffering varying widely depending on a multitude of factors, including their biology, degree of legal protection, the purpose of their trade, and how they are perceived by humans [11,12]. For instance, some animals are captured and killed, whereas others may be captive-bred and traded for life in captivity [11]. At each stage of trade, the intrinsic value and sentience of wild animals are often disregarded, and their well-being is largely unprotected by legislation [13]. As a result, billions of animals endure poor welfare throughout all stages of the trade [11,14]. Furthermore, despite the growing awareness of animal sentience across the range of taxa, including mammals, birds, amphibians, fishes, and invertebrates [15,16,17,18,19,20,21,22,23], and the negative impacts of trade on animal welfare, this area has received relatively little research attention.

To address this and to explore the diverse experiences of individual animals in the commercial wildlife trade, we have chosen ten case studies from various taxa and industries to represent the scale and breadth of the commercial wildlife trade: (1) ball pythons (*Python regius*), zebrafish (*Danio rerio*) and African grey parrots (*Psittacus erithacus*) traded as pets; (2) frogs (Anura) and crickets (Orthoptera) traded for food consumption; (3) pangolins (Pholidota) and sharks (Elasmobranchii) traded as traditional medicine; (4) crocodiles (Crocodylia) farmed for fashion; and (5) lions (*Panther leo*) and elephants (*Elephas maximus* ssp.) used for entertainment. For each of these case studies, we document the number of individuals involved and the process experienced by the animal to allow an assessment of the associated welfare impacts, their extent, and scale. The authors would like to alert readers that some individuals may find the content of this research to be distressing, in particular, given that we have included photographic evidence for each case study.

Our review comes at a critical juncture because the commercial wildlife trade continues to grow, and global demand expands across diverse species [6]. Economic growth in some areas and the influence of social media trends have likely contributed to commercial wildlife trade becoming more desirable [24,25,26]. In turn, the rapid rise in online platforms has further accelerated this trend, dismantling traditional barriers to trade and enabling the commodification of countless wild animals on a global scale [27,28].

## 2. Methods

For each of the case studies, we explore three different metrics: (1) the degree of welfare compromise experienced by the animals during each stage of the process, (2) the duration of each phase of the trade, and (3) the estimated number of animals traded. These indicate the extent of the impact, duration, and scale (i.e., how many individual animals are affected).

Accurate information on the conditions experienced over the entire trade chain and the number of animals involved is typically unavailable; therefore, we have drawn on various sources, from trade reports to scientific publications, to determine the most inclusive and objective figures possible. To assess the welfare impacts in each case study, we systematically reviewed the trade process from initial capture or breeding to eventual use or consumption, considering each stage from the animal’s perspective. This approach allowed us to identify key welfare concerns at different points in the trade chain.

We used the Five Domains Model [29] to assess the degree of welfare compromise an animal will likely experience at each trade process stage. The Five Domains Model is a credible and adaptable framework for assessing animal welfare, making it ideal for evaluating the variety of contexts encountered in the wildlife trade. The Model has been widely used and extensively applied across various mediums, including policy, legislation, and certification schemes [30,31]. The Model focuses on the mental state of animals and defines animal welfare as the balance between an animal’s positive and negative experiences and feelings [29]. The Model assesses the animals’ experience in accordance with their internal states and external influences under each of four domains (Nutrition, Environment, Health, and Behaviour) and then uses these to assess their overall Mental State (Domain 5) [30]. The Five Domains Model is particularly suited for use in this review as it places the emotional experience of the animal at the forefront of the assessment, rendering it a suitable means for assessing the welfare compromises an animal may experience due to commercial trade, especially when considering the implications of differing durations of experiences.

The case studies were chosen based on the authors’ expertise and to provide a broad overview of the welfare issues and experiences of animals traded for different purposes and across different taxa. We acknowledge that some of the species included are traded for multiple purposes (e.g., zebrafish are sold as pets and used in research [32], and ball pythons may be bred for pets, leather, or traditional medicine [33,34]. However, we intended to review animal experiences across various industries and not provide an exhaustive account of all possible species’ uses. In addition, our intention was not to identify the worst offenders when selecting these case studies but rather to showcase some concerning welfare issues in the predominantly legal wildlife trade. Accordingly, we recognise that even more concerning examples may exist.

### Definitions

The following case studies use specific terms that may be viewed differently depending on the stakeholder. Therefore, for clarity, we define the most pertinent terms below. We define commercial wildlife trade as the exploitation of wild animals for financial gain that does not include trade for subsistence purposes [35]. In contrast, we consider subsistence use as situations where wild animals are hunted for personal consumption by the hunter, their family or their local community [36]. However, such use is not the focus of this review. Instead, the focus of this review is on the “consumptive commercial wildlife trade”, which we define as the deliberate exploitation, collection, or killing of wild animals for commercial gain through activities, including trophy hunting, captive wild animal breeding, and the commercial (non-essential) use of wild animals for protein or traditional medicine consumption. The term consumptive applies to trade that involves killing the animal or, conversely, taking a live animal from the wild for other purposes, such as the pet trade. We define a wild-animal farm as a facility that breeds, rears, and potentially slaughters wild animal species rather than traditional agricultural animals [37]. A ranch is defined using the CITES definition; “rearing in a controlled environment of animals taken as eggs or juveniles from the wild …” [38]. An animal is ‘wild-caught’ when taken directly from the wild and is ‘captive-bred’ when bred in captivity.

## 3. Case Studies

### 3.1. Ball Pythons Captured and Ranched for the Pet Trade

Ball pythons (*Python regius*) are one of the most highly traded reptiles in the world [34] and are the most common CITES-listed species exported from Africa [39]. Around 100,000 live ball pythons are legally exported yearly from West Africa for the pet trade [39,40]. Over 90% of these are declared as “ranched” [39], and exports of reportedly captive-bred animals are also increasing [39,40,41].

Hunters typically dig pythons out of their burrows, which is likely stressful for the snakes involved and can cause physical injuries and mortality (compromising Domains 3 and 5) [40,42]. Eggs may be collected for ranching, but gravid females may also be collected to be bred in captivity [39,42]. Captured individuals are restrained alive in sacks with other snakes [42] before being transferred to holding facilities (that are typically unhygienic, crowded, and hot, as well as lacking veterinary care and disease protocols [41] (compromising Domains 1–5).

When ranched, a proportion of the hatched juveniles, and potentially also females who have laid eggs, are released back into the wild [41,43]. However, little attention is paid to where they originated from or whether the release site has a suitable habitat, which can result in mortality and genetic pollution [39,42,43].

The snakes for export are then typically transported in bags and in close confinement and proximity to other snakes on journeys that can last from hours to weeks to consumer countries in North America, Asia, and Europe (compromising Domains 1–5) [34]. International transportation is associated with high mortality rates (as high as 33% for reptiles in general) due to poor conditions (e.g., hot, crowded) often experienced for long periods (compromising Domains 1–5) [42,43,44,45]. Captive ball pythons experimentally exposed to handling and confinement have been shown to exhibit significant increases in plasma corticosterone, indicating an anti-predator stress response (compromising Domains 4 and 5) [46].

**Figure 1:**
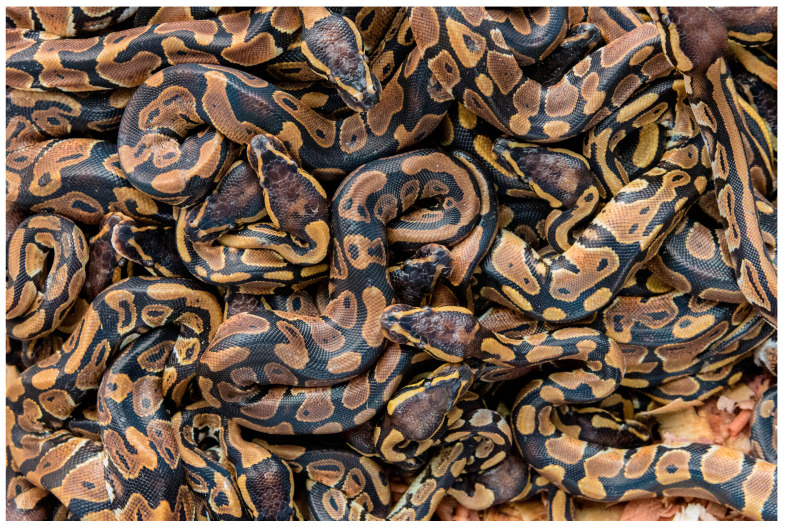
A mixture of wild-caught, ranched, and captive-bred ball pythons intended for the international commercial pet trade. Credit: Neil D’Cruze/World Animal Protection.

It is then commonplace for ball pythons to be held by traders in small and highly restrictive enclosures [34] where they cannot exercise, move adequately, or stretch out fully (compromising Domains 2, 4, and 5) [34,47] (Figure 1), and experience high mortality rates [16]. Ball pythons may then be kept as pets for weeks to years, often in barren and inappropriate environments (compromising Domains 2, 4, and 5), where they are also subject to interactions with humans that can negatively impact their well-being (compromising Domains 4 and 5) [46]. Furthermore, reptile pet owners commonly mistake signs of stress and poor welfare as being ‘normal’ (e.g., rapid open-mouth breathing and high levels of boundary exploration) (compromising Domains 3–5) [20,48,49]. This may be why mortality rates of reptiles can range from 3.6 to 75% for reptiles in their first year of captivity [50,51].

Collectively, the welfare compromises seen across the trade chain and throughout the lives of ball pythons captured and reared for the pet trade negatively impact the Fifth Domain: Mental States, with a range of potential negative affective states, including thirst, hunger, discomfort, pain, stress, fear, frustration, boredom, sickness, stress, exhaustion, and distress (see Table 1 and Table S1).

### 3.2. Zebrafish Captive-Bred for the Pet Trade

**Figure 2:**
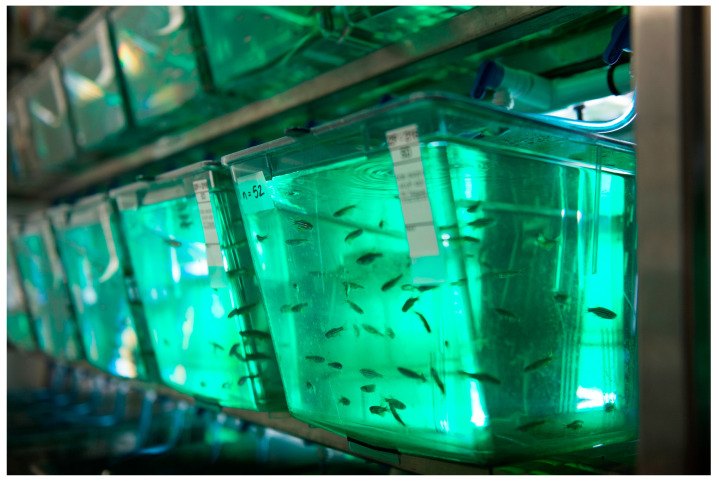
Captive-bred zebrafish intended for the international commercial pet trade. Credit: “NIH Zebrafish Facility” by National Institutes of Health (NIH), licenced under CC BY-NC-2.0.

Although the scale of the trade in pet zebrafish (*Danio rerio*) is unknown, over five million are thought to be bred for research each year [52]. The treatment of zebrafish in aquaria has been described as misguided and inadequate, compromising each of the Domains [53,54]. For example, zebrafish are typically bred in crowded, barren tanks with no enrichment (Figure 2), resulting in signs of boredom and the performance of stereotypical behaviours (compromising Domains 2, 4, and 5) [55,56].

Zebrafish can also suffer from increased stress, morbidity and mortality, and decreased affiliative behaviours when exposed to husbandry stress (e.g., poor water quality) and husbandry practices (e.g., water changes) (compromising Domains 2–5) [57,58]. These effects may last for days, months, or even years (see Table 1 and Table S2).

**Table 1:** The experiences of ball pythons *Python regius*, zebrafish (*Danio rerio*), and African grey parrots (*Psittacus erithacus*) in the commercial wildlife trade for exotic pets, presented in three metrics; the estimated numbers of animals involved, the potential duration of their experiences for each phase of the trade, and the severity of their welfare compromises, described using the Five Domains model.

				Severity of Welfare Compromises				
Species	Numbers	Trade Stage	Duration	Nutrition	Environment	Health	Behaviour	**Mental States**
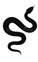	100,000 exported live from West Africa/yr [39,40].	**Capture**	Hours to days	Restricted water and food intake; inability to hunt; inadequately sized water containers	Thermal extremes likely; close confinement; unpredictable events/noises; barren environment and inadequate stimulation	Risk of disease, injury, and suffocation from overcrowding; high morbidity and mortality rates	Barren and inappropriate environment; no control; significant constraints on behaviour; negative interactions with humans	Thirst, hunger, discomfort, pain, stress, fear, sickness, stress, exhaustion and distress
		**Transport**	Hours to weeks	Restricted water and food intake; inability to hunt; inadequately sized water containers	Thermal extremes likely; close confinement; unpredictable events/noises; barren environment and inadequate stimulation	Risk of disease, injury and suffocation from overcrowding; high morbidity and mortality rates	Barren and inappropriate environment; no control; significant constraints on behaviour; negative interactions with humans	Thirst, hunger, discomfort, pain, stress, fear, sickness, stress, exhaustion and distress
		**Pet Trade**	Weeks to years	Inability to hunt; inadequately sized water containers	Thermal extremes likely; close confinement; unpredictable events/noises; barren environment and inadequate stimulation	Potential for ill health and mortality dur to poor husbandry	Barren and inappropriate environment; no control; significant constraints on behaviour; negative interactions with humans	Discomfort, pain, stress, fear, boredom, sickness, stress, and distress
		**Pet Ownership**	Weeks to years	Inability to hunt; inadequately sized water containers	Unpredictable events/noises; barren environment and inadequate stimulation	Potential for ill health and mortality dur to poor husbandry	Barren and inappropriate environment; no control; significant constraints on behaviour; negative interactions with humans	Discomfort, pain, stress, fear, boredom, sickness, stress, and distress
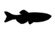	Unknown (over 5 million are bred for research/yr) [52].	**Captive breeding**	Days to weeks (breeder; 2–3 years)	Unnatural feed delivery; no control over feeding schedule	Continual changes to environment; risk of poor water quality; unpredictable events/noises; barren/unstimulating tanks	risk of disease; risk of painful injuries from netting and aggressive individuals; risk of poor health from mismanaged water quality; risk of husbandry stress; mutilations including dye-tattoos and injections	Barren and restricted environment; lack of control; significant behavioural constraints	Discomfort, pain, stress, fear, sickness, boredom, frustration, and distress
		**Transport**	Hours to days	Feed withdrawal	Severe confinement; risk of thermal extremes; unpredictable events/noises	Risk of disease, injuries, poor health, and husbandry stress	Barren and restricted environment; lack of control; significant behavioural constraints	Hunger and exhaustion
		**Ownership**	Weeks to years	Unnatural feed delivery; no control over feeding schedule	Continual changes to environment; risk of poor water quality; unpredictable events/noises; barren/unstimulating tanks	Risk of disease; risk of painful injuries from netting and aggressive individuals; risk of poor health from mismanaged water quality; risk of husbandry stress; mutilations including dye-tattoos and injections	Barren and restricted environment; lack of control; significant behavioural constraints	Discomfort, pain, stress, fear, sickness, boredom, frustration, and distress
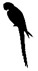	~1.3 million CITES recorded exports since 1975 (the figure does not account for much of the concealed illegal trade) [59,60].	**Capture**	Hours to days	Restricted water and food intake	Thermal extremes likely; close confinement; unpredictable events/noises; barren environment and inadequate stimulation	Risk of disease from close confinement and crowded housing; risk of painful injuries from capture methods, including feather damage from glue traps, and injuries from nets; young chicks are often unable to survive independently	Significant constraints on behaviour for duration of capture, including being unable to move, feed, or drink; Negative interactions with humans	Hunger, thirst, exhaustion, discomfort, pain, stress, fear, sickness, frustration, and distress
		**Transport**	Hours to days	Restricted water and food intake	Thermal extremes likely; close confinement; unpredictable events/noises; barren environment and inadequate stimulation		Barren and inappropriate environment; no freedom to make choices; significant constraints on behaviour for long periods, including being unable to move, perch, stretch wings, feed, or drink; negative interactions with humans	Hunger, thirst, exhaustion, discomfort, pain, stress, fear, sickness, frustration, and distress
		**Ownership**	Weeks to years	Inappropriate diets; unnatural feed delivery; no control over feeding schedule	Close confinement compared with natural range; unpredictable events/noises; unnatural and inadequate stimulation		Inappropriate environment; No freedom to make choices; Significant constraints on behaviour for long periods; Potential for positive or negative interactions with humans	Hunger, thirst, discomfort, pain, stress, fear, sickness, boredom, frustration, and distress

Stressful experiences during transportation (e.g., from nursery and breeding sites to traders and consumers, which can last from hours to days) include handling, unloading, confinement, regrouping, and unpredictable events, sounds, temperatures, and movements (compromising Domains 1–5) [61,62]. Zebrafish are typically transported in plastic bags with no filtration, sometimes for extended periods, which can cause prolonged increases in stress levels and may also be associated with increased rates of sickness and mortality (compromising Domains 1–5) [53,61].

Zebrafish are subject to a range of diseases in captivity (e.g., aquatic viruses such as the haemorrhagic septicaemia virus and various fungal infections), many of which cause significant pain and suffering (compromising Domains 3 and 5) [63,64]. The mortality rates of zebrafish in trade are unknown, and estimates are strongly contested for pet fish in general [53]. For instance, estimated ranges of mortality as a result of transportation and handling processes range anywhere from 2% to 73%, depending on species, conditions, and how numbers are calculated [53,65,66,67,68]. High mortality rates may be due to keeper ignorance regarding husbandry needs and/or failure to seek veterinary care (compromising Domains 1–5) [53,54], which may be explained by general neglect of fish welfare and a lack of recognition of their sentience [18,69].

As a result of the welfare compromises that zebrafish experience across the Domains, the fifth Domain, Mental States, is likely to be adversely impacted, leading to negative affective states, including hunger, exhaustion, discomfort, pain, fear, stress, sickness, boredom, frustration, and distress (see Table 1 and Table S2).

### 3.3. African Grey Parrots Captured for the Pet Trade

**Figure 3:**
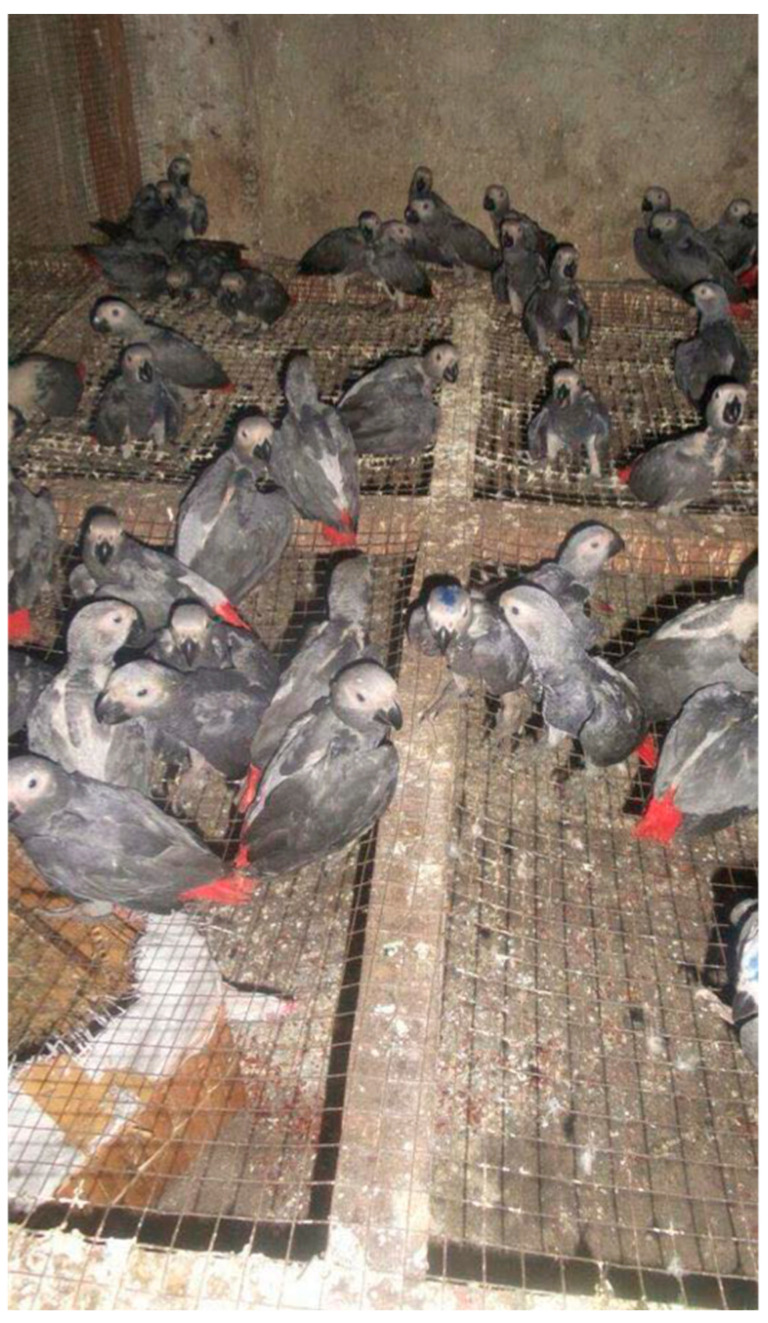
African grey parrots during international transport for the pet trade. Credit: Anonymous.

African grey parrots (*Psittacus erithacus*) are traded for food, medicine, and the international pet trade, the latter being the most significant in terms of the number of animals involved [70,71]. In 2016, when Grey parrots were listed in CITES Appendix II, over 1.3 million wild-caught Grey parrots had been exported from 18 range States since 1975 [59,72]. This makes the Grey parrot the most traded of all CITES-listed birds, representing 11% of all reported parrots in the wildlife trade [59,72]. Due to concerns over the rapid decline in wild populations and the facilitating role of trade, Grey parrots were listed in Appendix I of CITES in 2017, preventing wild-caught individuals from being traded commercially. However, whilst captive-bred parrots now dominate the trade [73], illegal trafficking continues, with individuals often concealed in exports of other unprotected parrot species [74], resulting in considerable welfare implications (see Figure 3) [71,74,75].

Methods of trapping parrots in the wild range from taking chicks from nest cavities to mass trapping using nets or glue traps (compromising Domains 1–5) [76]. The latter includes the glue-and-stick method, where broomsticks or branches coated with plant sap trap birds’ wings as they land to roost or feed [75]. Fishing nets are also used to entangle birds at roosting or feeding sites, where the birds are chased into the nets [75]. These methods will likely cause extensive suffering to the birds across all Domains [75].

Mortality rates due to these trapping methods are likely to be impossible to quantify with precision due to the variation in methods used, the experience of the hunters, and the illegal nature of the activity, and estimates can range from 30 to 66% [71,75,76,77,78,79]. Transportation may result in more mortalities, with one study reporting 9–14% mortality rates between the forest and the trappers’ homes [75]. These high mortality rates are often the result of hunters taking chicks who are too young to survive independently, but the stress and physical trauma of the capture process, and the overcrowding, physiological stress, lack of food, water, and veterinary care throughout transportation also play a role (compromising Domains 1–5) [71,75]. In fact, analyses of social-media listings of African Grey parrots suggest that basic animal welfare standards were frequently breached during transportation and in holding facilities, with parrots being kept in overly crowded conditions, with no perches, and infrequent or no provision of food and water for days or even weeks (compromising Domains 1–5) [72].

Therefore, the trade in African Grey parrots adversely compromises the welfare of parrots across the Domains, leading to a range of negative affective states, including hunger, thirst, pain, fear, distress, sickness, frustration, stress, and exhaustion (see Table 1 and Table S3).

### 3.4. Sharks Wild-Caught for the Shark Fin Trade

International trade of almost all shark species traded for their fins has been regulated under CITES since CoP19 [59]. However, domestic (local) trade and illegal trade may remain due to a lack of meaningful action, particularly in major import hubs where enforcement measures are insufficient [80,81]. It has been conservatively estimated that around 63 to 273 million sharks and rays (Class: Chondrichthyes) are killed each year for the shark fin trade [82]. According to the FAO Global Fish Trade Statistics Database, in 2020, 12,391 tonnes of shark fins were imported globally [83]. However, given the illegal nature of much of the trade, these numbers are considered gross underestimates [84,85], with the true scale of the shark fin trade estimated to be around three or four times higher than the FAO estimates [84].

**Figure 4:**
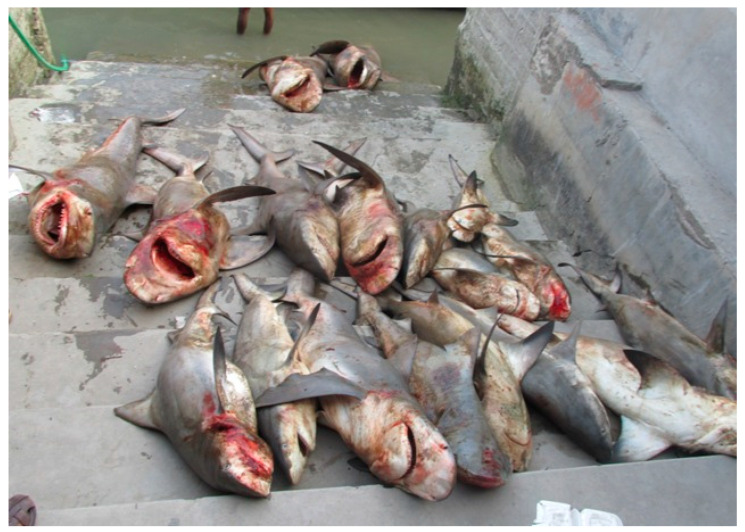
White cheek shark slaughtered for the shark fin trade by USFWS Headquarters, Public Domain Mark 1.0.

Most sharks are caught (incidentally or targeted) using longlines [86], which can cause stress and tissue damage (compromising Domains 1–5) [87,88]. For instance, juvenile tiger sharks (*Galeocerdo cuvier*), released after being caught on a long line, exhibited changes in behaviour indicative of stress and avoidance learning (compromising Domains 4 and 5) [89]. Once captured, sharks are hauled on board, sometimes by stabbing with a ‘gaff’ (long pole and hook) (compromising Domains 2–5), to have their fins sliced off, a process that can last from seconds to minutes [90,91]. During this time, the sharks are typically conscious and exposed to considerable pain and distress (compromising Domains 3 and 5) [18,90,92,93] (see Figure 4).

Due to the high value of fins and the illegal nature of much of the trade [94,95,96], many de-finned sharks are thrown back into the sea alive to die slowly and painfully because they can no longer swim and either bleed out, asphyxiate or are predated on by other fish (compromising Domains 1–5) [92,93,97].

Suffering for these species can last from minutes to days, and there are welfare compromises across the Domains. Overall, sharks killed for their fins are likely to experience a number of negative affective states, including hunger, pain, discomfort, fear, stress, exhaustion, frustration, and distress (see Table 2 and Table S4).

**Table 2:** The experiences sharks and pangolins in the commercial wildlife trade for traditional medicine, presented in three metrics; the estimated numbers of animals involved, the potential duration of their experiences for each phase of the trade, and the severity of their welfare compromises, described using the Five Domains model.N/A: Not applicable.

				Severity of Welfare Compromises				
Species	Numbers	Trade Stage	Duration	Nutrition	Environment	Health	Behaviour	Mental States
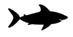	~63–273 million sharks/yr (estimated calculation, although likely to be more when including illegal trade) [82].	**Capture**	Hours to days	Restricted food intake	N/A	Injury from longline hook or net, and tools and landing procedure	Severe restriction of behaviour	Hunger, pain, discomfort, fear, stress, exhaustion, frustration, and distress
		**Fin removal**	Seconds to minutes	N/A	N/A	Severe injury and pain	Severe restriction of behaviour post fin-removal; Negative interactions with humans	Pain, discomfort, fear, stress, and distress
		**Killing**	Seconds to hours	N/A	N/A	Slow and inhumane death from suffocation, predation, exsanguination, or asphyxiation	Negative interactions with humans	Pain, discomfort, fear, stress, and distress
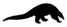	~195,000 in 2019 (based on intercepted trade so the actual figure is likely to be greater) [98].	**Capture and Transportation**	Hours to weeks	Restricted food and water intake	Thermal extremes likely; highly restricted confinement with absence of light and fresh air; unpredictable events and noises; barren environment	High risk of disease; high risk of painful injuries from capture methods; potential for smoke inhalation, burns; dog bites and trap and spear injuries; risk of severe cramping from being unable to uncurl for long periods	Barren and inappropriate environment; lack of control; significant constraints on behaviour, including being unable to unfurl; negative interactions with humans	Extreme thirst and hunger, discomfort, pain, stress, fear, sickness, frustration, boredom, exhaustion, and distress
		**Killing**	Seconds to hours	Restricted food and water intake	N/A	Inhumane, slow, and painful death, including the potential for conscious individuals to be boiled alive	Negative interactions with humans	Pain, discomfort, fear, stress, and distress

### 3.5. Pangolins Trapped and Killed for Traditional Medicine

All nine extant species of pangolins in Africa and Asia are illegally traded internationally for their meat and scales [98,99,100]. Despite being listed in Appendix I of CITES in 2016 and banned from international commercial trade, pangolins are often cited as the most heavily trafficked CITES-regulated mammal [33,100,101,102]. Pangolin scales are used in traditional medicines (TM) and have a high financial value, with reports of a kilo of pangolin scales being worth USD 650 in 2019 [103]. Pangolin meat is also considered a delicacy in some countries, particularly China and Vietnam [26,104], and is believed by some to have healing properties [98,105].

In 2019, around 195,000 pangolins were known to be trafficked, although as this is based on the portion of illegal trade that is successfully intercepted, it may be an understatement [98]. Furthermore, the focus in recent years has moved to the more abundant African and Indian pangolin species, which has meant that the illegal trade has continued to occur and even increased in some areas despite decreasing numbers of pangolins in the wild [98,106,107,108,109,110].

Hunting methods include opportunistic captures, tracking with dogs, digging, felling or burning trees, smoking out dens, and setting traps like snares (compromising Domains 1–5) [105,111,112,113,114]. Escaping pangolins may be caught by hand or spear (compromising Domains 3–5) [105].

**Figure 5:**
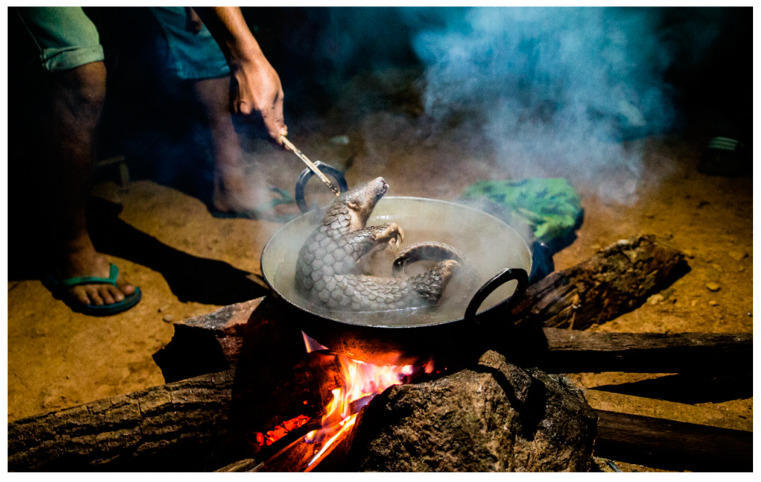
A wild-caught pangolin destined for the traditional medicine trade. Credit: Neil D’Cruze/World Animal Protection.

Once caught, the pangolins are usually tightly tied in individual netting sacks before being killed or traded alive (see Figure 5) (compromising Domains 1–5) [112].

There are also reports of pangolins being force-fed with cement and plaster to increase their body weight and, therefore, value [115]. Pangolins are typically killed by blunt force or cut on the head and then boiled for easier scale removal [105]. However, as these methods are not reliable, some individuals may still be alive when they are put in boiling water, resulting in extensive pain and suffering (compromising Domains 3 and 5) [105].

A pangolin may be pursued and hunted for hours or even days and may experience distress, fear, pain, and suffering throughout (compromising Domains 3 and 5) [105]. For example, pangolins caught by spears or snares or hunted by dogs are likely to be wounded and in pain. Indeed, dog bite wounds around the base of the tail are commonly seen in confiscated pangolins (compromising Domains 3 and 5) [116].

For pangolins traded alive, transportation methods compromise several welfare domains [116]. For example, confiscated pangolins are often found tied tightly in their defensive balled position in net sacks and stacked upon one another [116]. Such methods prevent the provision of water and food and deny the pangolins the chance to move or even to uncurl (compromising Domains 1–5). The proximity of other pangolins may also be stressful and potentially painful if the animals are crushed or suffocated (compromising Domains 2–5). Hygiene is reportedly low in these shipments, as evidenced by confiscated pangolins being covered in faeces and urine and suffering from a range of infectious diseases and parasites (compromising Domains 2, 3, and 5) [116]. Injuries from the hunting process and transportation are also commonly seen in confiscated pangolins, and individuals are often infected at the point of confiscation, which can result in loss of limbs or fatal septicaemia (compromising Domains 3 and 5) [116].

Collectively, the welfare compromises that pangolins experience during capture, transport, and killing are considerable, and they are likely to experience several negative emotional states, including extreme thirst and hunger, discomfort, pain, stress, fear, sickness, frustration, exhaustion, boredom, and distress (see Table 2 and Table S5).

### 3.6. Crickets Killed for Food and Feed

**Figure 6:**
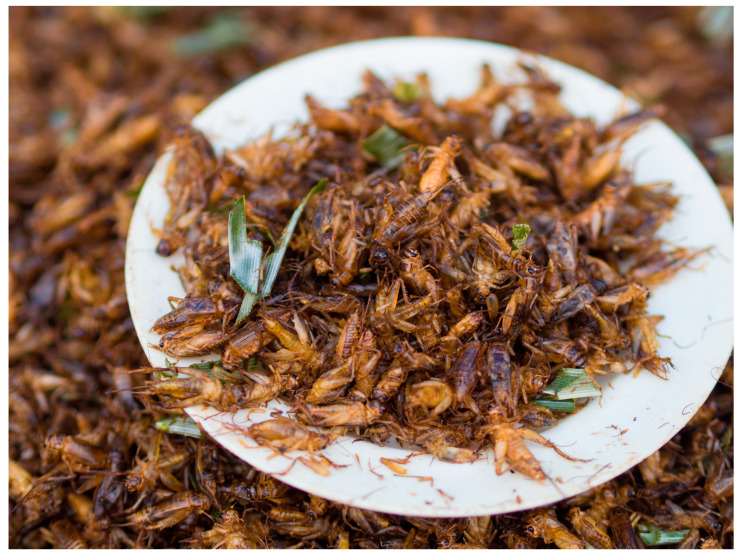
Commercially farmed crickets intended for human food products and livestock feed. Credit: “IMG_6583_01” by killerturnip, licenced under CC BY-NC ND 2.0.

Globally, insect farms are increasing in size and number, driven by demand for a range of commercial products, including cereal bars, flour, snacks, and livestock feed [117] (see Figure 6). Consumption of edible insects, including crickets (*Gryllidae*), as a source of human and animal protein has been promoted by the United Nations (UN) Food and Agriculture Organisation (FAO) since around 2013 [118]. In 2020, 370 billion–430 billion crickets (typically the house cricket, *Acheta domesticus*) were estimated to be sold or killed per year for the trade, and an average of 34 billion–41 billion crickets were estimated to be alive on farms at any one time [119].

Despite the numbers involved, there is a significant lack of understanding of the welfare implications of this trade for the invertebrates involved [19,120,121]. A growing body of research suggests that there may be a case for considering crickets as sentient beings, highlighting the need to review current farming practices [19,122,123]. For example, crickets are thought to be capable of mental stress and the cognitive abilities of decision-making, learning, recognition, and long-term memory [19].

There has been considerable trial and error in developing large-scale cricket farms, with mistakes resulting in millions of cricket mortalities [119,124,125]. For example, crickets reared on “waste streams” (e.g., manure or urban and catering waste) suffer high premature mortality rates (>99%) (compromising Domains 1, 3, and 5) [125]; overcrowding facilitates the spread of disease, compromises the environment, and restricts their behaviour (compromising Domains 2–5) [126]; and the lack of veterinary knowledge regarding insects or the pests and diseases that affect them further impacts their survivability and welfare (compromising Domain 3) [127]. In addition, the lack of evidence regarding best practices for slaughter methods represents a potential welfare concern [128], especially as some of the methods currently used, including boiling, are known to cause suffering (i.e., physiological shock and behavioural signs of aversion) to other invertebrate species, such as decapod crustaceans [129].

The welfare compromises that farmed crickets can experience across the Domains have the potential to give rise to negative mental states, including hunger, thirst, discomfort, pain, stress, fear, sickness, boredom, and frustration (see Table 3 and Table S6).

### 3.7. Frogs Wild-Caught for the Frogs’ Leg Meat Trade

The commercial trade in frogs’ legs is estimated to involve 81–200 million frogs (*Anura*) annually [130]. Most frogs in the frogs’ leg trade are wild-caught [131]. Large-scale commercial farming for the trade has also steadily increased in recent years, but wild-caught individuals are collected to restock farms [35,132]. Frogs’ legs are consumed globally, but demand is greatest in Western Europe [130]. The European Union alone imported around 40,700 tonnes of mainly wild but also farmed frogs’ legs between 2011 and 2020 [131]. The imports came from several source countries, including Indonesia, Vietnam, Turkey, and Albania [130]. Some frog species are also sourced domestically in Europe; for example, >2 million common frogs (*Rana temporaria*) are legally caught every year in France for trade [130]. Overall, though, the North American bullfrog (*Aquarana [Lithobrates] catesbeiana*) is the most farmed amphibian globally [132], primarily in Taiwan, with considerable contributions from countries like Ecuador and Brazil [133].

Wild-caught frogs are typically processed within hours or days of capture, although live transport for consumption also occurs [131,134]. Hunters usually use a three-headed spear (trident) on a long pole or a net to capture wild frogs (compromising Domains 3 and 5) [135]. Frogs caught with a spear suffer a painful, frequently slow death, and many (at least 2–5%) are refused by exporters due to physical damage or bruising (compromising Domains 3 and 5) [135]. Frogs captured with a net are kept alive in overcrowded bags, buckets, or wire cages (Auliya, pers. obs.), often among different species and with severely limited space, air, and water, which increases the risk of disease transmission, suffocation, and crushing (compromising Domains 1–5) [131,136].

**Figure 7:**
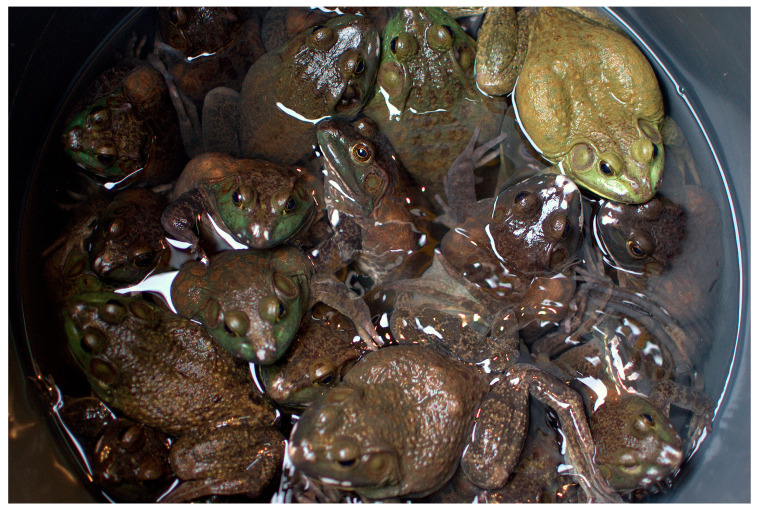
Wild-caught frogs intended for human consumption as food. Credit: “Frogs for sale” by caperry123, licenced under CC BY-ND 2.0.

Subsequent transportation of live frogs subjects them to overcrowded and confined conditions where they cannot engage in natural behaviours, including feeding, moving, or resting (Domains 1–5) [131,134] (see Figure 7). Many frogs are dead on arrival at processing plants and cannot be exported [134]. Overall, premature mortality rates for the international frogs’ leg trade are unknown, but one study published more than 35 years ago reported pre-export mortalities to be 10–20% [137].

At the market or processing plant, live frogs typically have their legs removed whilst conscious, either by cutting with scissors or a knife or dismemberment by hand, without pain relief (compromising Domains 3 and 5) [131,134]. Death can take from seconds to hours (compromising Domains 3 and 5) [17]. In total, trade-related welfare impacts last from hours to weeks (see Table 3). No welfare regulations are currently in place for the frogs’ leg trade, despite the considerable volume of imports to the EU [131], as well as recent education campaigns (e.g., [138]) and updated welfare standards and certification (e.g., [139]).

The experience of frogs traded for the frog leg market can compromise welfare across the Five Domains, leading to a range of negative mental states, including thirst, hunger, discomfort, pain, fear, stress, sickness, frustration, exhaustion, and distress (see Table 3 and Table S7).

### 3.8. Crocodilians Farmed for Their Skins

Crocodilians (Crocodylidae) have been commercially farmed for their skins (and meat as a by-product) since the 1970s [140]. According to the International Union for the Conservation of Nature (IUCN) Crocodile Specialist Group, over 1.5 million crocodilian skins are legally exported annually from around 30 countries [141] and 5000 crocodilian farms worldwide [140]. The saltwater crocodile (*Crocodylus porosus*) is a popular species for farming and ranching in Australia due to their large size and high-quality skin [141]. The species is also widely farmed in Papua New Guinea and Indonesia, and many range States of *C. porosus* in South and Southeast Asia and the Pacific maintain farming facilities [142,143].

**Figure 8:**
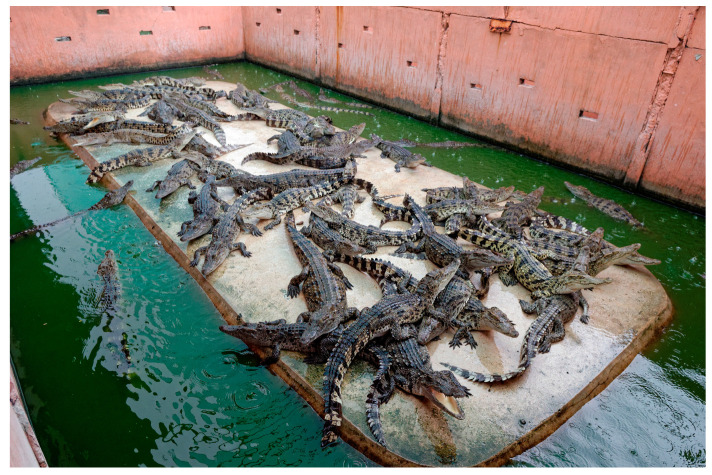
Captive-bred crocodiles farmed for their skins and meat. Image from 2016, Credit: Jan Schmidt-Burbach/World Animal Protection.

Like other crocodilians, saltwater crocodiles are sentient, wild animals with innate drives, behaviours, and mental needs [20,144]. The species is territorial and competitive and can develop co-occupant aggression when close to others, and more dominant individuals guard important resources such as water [144,145,146]. Despite this, saltwater crocodiles are typically held in groups until around 1–2 years of age with limited space and access to water (densities vary with age from around 0.07 m^2^/animal for hatchlings and 0.28 m^2^/animal for yearlings [147] (compromising Domains 2, 4, and 5) [144,146,148]. Because saltwater crocodiles are predominantly farmed for their belly skin, they are kept in individual pens for the final stage of production (several months to a year) to avoid imperfections (e.g., scratches) on their belly [148], which is likely to severely restrict movement and natural behaviour (compromising Domains 2, 4, and 5) (see Figure 8).

Farming crocodilians has been a learning curve, and in the first decade of farming, mortality rates were over 30% and even higher during the dry season [149]. With improvements, mortality rates have dropped to between 1.6% and 0.98%, although hatchling mortalities remain high (13.4%) [144]. Captivity-related stressors such as thwarted innate drives, overcrowding, social disruption, maladaptation, handling and restraint, noise, poor hygiene, and poor diet lead to physiological, behavioural, and mental compromises, including immunosuppression, disease susceptibility, obesity, injuries, and infections (compromising Domains 1–5) [150,151,152,153]. Intensive rearing can also trigger disease outbreaks (e.g., Crocodile pox, Chlamydiosis, Salmonellosis) and mortality, particularly due to thermoregulation issues (compromising Domains 2, 3, and 5) [144,151,154].

Slaughter methods also pose welfare concerns, as the IUCN Crocodile Specialist Group recommends brain destruction for slaughter [141]. However, investigations in Australian farms have found crocodiles may still be ‘processed’ whilst still alive (compromising Domains 3 and 5) [155,156]. However, the IUCN Crocodile Specialist Group disputes this, stating that crocodilians may continue to move due to “spontaneous muscle spasms within tissues” when their brains have been effectively destroyed [157]. Nevertheless, it does not appear to be common practice for operators to ensure unconsciousness before processing individuals, which may mean that some individuals are sensible to pain and suffering as a result (compromising Domains 3 and 5) [158,159,160,161,162].

The significant welfare compromises that crocodilians experience throughout the farming process negatively impact each of the Domains and potentially give rise to the following negative affective states: hunger, frustration, stress, distress, pain, discomfort, and pain (see Table 4 and Table S8).

### 3.9. Lions Farmed for Tourist Attractions

Commercial lion (*Panthera leo*) farming is a significant and growing industry in South Africa, with around 8500 lions estimated to be registered at nearly 400 facilities across the country, contributing around USD 42 million annually to the South African economy [163,164]. The lions are destined for either tourism industries, including cub petting, voluntourism, and ‘canned’ hunting, or to have their body parts, particularly their bones, sold as by-products [163,165,166,167]. For example, in a survey of lion industries in South Africa, 72% (84) facilities reported they had sold lion products, including trophies, bones, or other parts, and 66% (77) had sold skeletons to South African lion bone traders [167].

**Figure 9:**
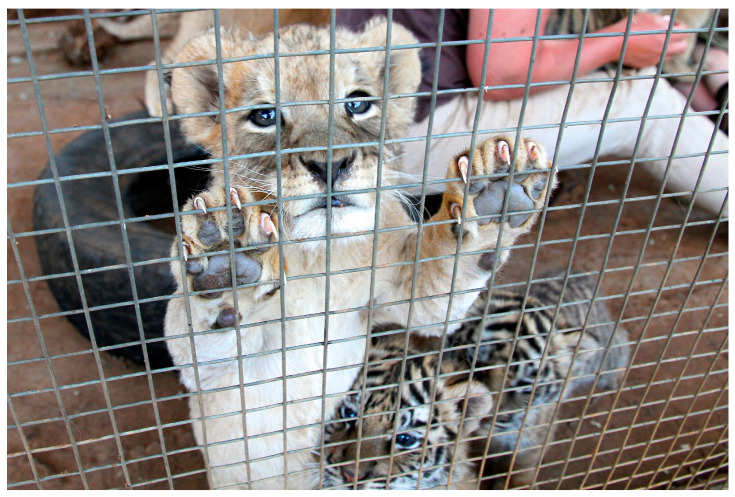
Captive-bred lion and tiger cub offered to tourists for petting and interactions. Credit: Pippa Hankinson/Blood Lions.

Lion farms are associated with numerous welfare concerns. Inspections by South Africa’s National Council of Societies for the Prevention of Cruelty to Animals (NSPCA) suggested that nearly half of the 95 facilities inspected had substandard conditions [168]. In particular, they found inadequate diets and malnourished lions, poor hygiene, including dirty water, a lack of proper veterinary care and treatment resulting in sick and injured animals suffering unnecessarily, along with numerous health issues, and small, barren, and overcrowded enclosures (see Figure 9), which resulted in bullying, severe injuries, and deaths (compromising Domains 1–5) [168].

The practices on lion farms are typically intensive and associated with many welfare concerns, including inbreeding, low reproductivity, poor maternal acceptance, increased cub mortality, and poor immune functioning (compromising Domains 1–5) [166,169]. Lionesses are often subjected to ‘speed breeding’ practices, where their cubs are removed before they are weaned, to encourage the female to return to oestrus [170]. This results in physical and psychological stress for the lioness and her cubs (compromising Domains 3–5) [166,169]. There is also a significant risk of increased disease outbreaks in these farms, including zoonotic diseases and pathogens (compromising Domain 3) [171].

**Table 4:** The experiences of crocodiles (Crocodylidae) in the commercial wildlife trade for fashion products (leather), presented in three metrics; the estimated numbers of animals involved, the potential duration of their experiences for each phase of the trade, and the severity of their welfare compromises, described using the Five Domains model.NA: Not applicable.

				Severity of Welfare Compromises				
Species	Numbers	Trade Stage	Duration	Nutrition	Environment	Health	Behaviour	Mental States
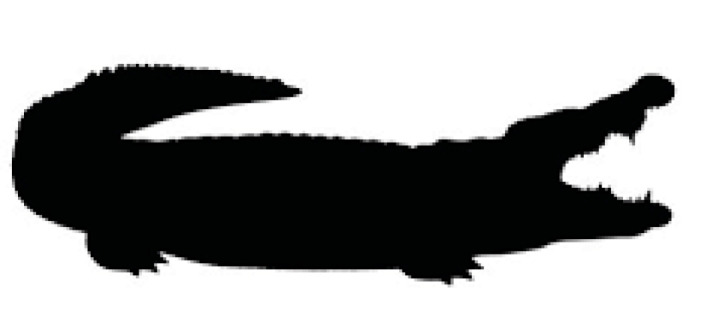	Numbers vary. 5000 crocodilian farms worldwide [140].	**Rearing**	2–3 years	Inappropriate diets/provision	Severe confinement in overcrowded, barren, unnatural environments.	Numerous diseases caused by captivity stress; crocodile pox, caimain pox, chlamydiosis, dermatophilosis, and salmonellosis.	Unnatural overcrowded groups for first phases. Inappropriate handling and restraint.	Hunger; frustration; stress; distress; pain; discomfort; fear
		**Killing**	Minutes–hours	N/A	N/A	Inhumane, painful killing methods, and potentially still conscious when being skinned.	Negative interactions with humans	Fear; pain; discomfort; stress

The cubs used for cub-petting and lion walking are susceptible to several specific welfare concerns. For example, cubs are subjected to unsupervised and forced handling by inexperienced humans, including children, from which they cannot escape (compromising Domains 2, 4, and 5) [169]. They may also be repeatedly separated from other cubs, ignoring their social needs and causing further distress (compromising Domains 4 and 5) [169]. These interactions result in fear and stress and may also be so frequent that they impact the cubs’ development by disturbing their rest and natural behaviour patterns (compromising Domains 3–5) [169]. Furthermore, because nutritional deficiencies are common in lion farms, excessive handling may also result in injuries and fractures because bone weaknesses are common, and the handling stress may also exacerbate their compromised immunity (compromising Domains 1, 3, 4, and 5) [168]. Larger cubs used for ‘walking with lion’ experiences are subject to disruption of normal behavioural patterns and lack free choice, and the lions are often required to walk on and off all day, even during high temperatures when they typically rest (compromising Domains 1–5) [172]. The lions must be trained to participate in this way, which may involve inhumane training practices (compromising Domains 4 and 5) [169,172]. When lions are too old for petting or walking activities, they may be sold for canned hunting or killed for the bone trade, both destinations being associated with numerous welfare concerns (compromising Domains 1–5) [169].

The experiences of lions used for tourist interactions compromise their welfare across the Domains. As a result, the lions may experience the following negative emotional states: hunger, thirst, trauma, discomfort, anxiety, pain, stress, fear, sickness, frustration, distress, boredom, and depression (see Table 5 and Table S9).

### 3.10. Elephants Used for Tourist Rides

Despite growing emphasis among travel trade associations to advocate for the protection of elephants from exploitation for tourism [173,174], direct tourist interactions with elephants are still commonplace and appear popular amongst tourists [173]. Across South and Southeast Asia, there are an estimated 3800 captive Asian elephants (*Elephas maximus*) kept in camps for tourists to ride and interact with [173]. Most of these elephants were captured from the wild [175], often after coming into conflict with people [176,177], or were former logging elephants [174]. Elephants typically remain in captivity for their entire working lives, which can be over 55 years [178].

**Figure 10:**
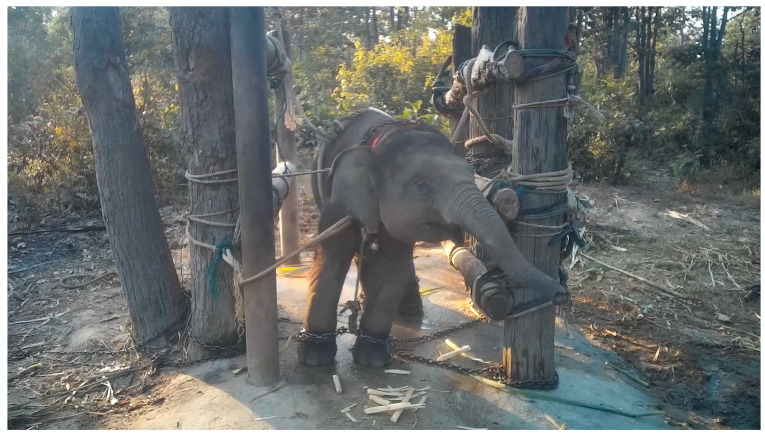
Captive bred elephant calf undergoing aversive conditioning to prepare them for tourism-related experiences such as elephant riding and circuslike performances. Credit: World Animal Protection.

Young calves are often separated from their mothers at 2–4 years old (compromising Domains 4 and 5) [173,179]. This causes considerable distress for the mother, who may be chained in her resting spot for up to two months to prevent her from searching for the calf (compromising Domains 2–5) [173]. Calves are then ‘broken’, typically by confining them and prodding and hitting them into submission, whilst they are tied down with chains that only allow minimal movement and sometimes prevent them from lying down (compromising Domains 1–5) [173] (see Figure 10). The training and isolation may last several days to two weeks [173,180]. This process causes intense pain, suffering, and exhaustion for these young animals, who will likely be in a heightened state of fear and distress after having been removed from their mother (compromising Domains 1–5) [181].

Elephants are then often trained using punishment and aversion-based methods, including using a hook (“ankush”), sticks, or nails to repeatedly scrape and apply pressure to sensitive points on the elephant’s body (e.g., the forehead or behind the ears) to incur pain (compromising Domains 3–5) [173]. This process can cause minor wounds, abrasions, major deep wounds, lacerations, ulcers, and abscesses (compromising Domains 3 and 5) [180,182]. ‘Positive training’ has been proposed by some to establish dominance through more subtle techniques (such as gradually increasing pressure on the elephant until they comply to avoid pain or discomfort) combined with rewards. This training may be less painful for the elephant, but it can still lead to psychological discomfort as it is still based on establishing dominance and does not eliminate punishment in cases of disobedience when the elephant is fully grown (compromising Domains 4 and 5) [173,180].

Typically, in elephant tourism facilities, elephants from different sources are housed together, with little opportunity to form social bonds that are a key component of their natural matriarchal family structures (compromising Domains 2, 4, and 5) [181]. The lack of social structure in captivity can negatively impact elephants, including elevated stress levels and the development of poorly adjusted individuals (compromising Domains 4 and 5) [183,184].

Most captive elephants in South and Southeast Asia are highly constrained in their movements (compromising Domains 2, 4, and 5), spending hours chained in one area and prevented from performing instinctive behaviours, such as foraging, bathing, and social interactions (compromising Domains 2, 4, and 5) [180]. This can considerably impact their psychological well-being (compromising Domain 5) [185]. Elephants in temples in southern India (and likely across Asia) are a case in point. Fed on inappropriate or restrictive food and chained their whole life, they are distressed, aggressive, and prone to severe intestinal stress (compromising Domains 1–5) [180]. Elephants who have experienced trauma, including separation from their mother, poor captive conditions, and painful training methods, can show signs of Post-Traumatic Stress Disorder (PTSD), even after they have been rehoused in sanctuaries (compromising Domains 2, 4, and 5) [186]. Other activities offered to tourists in some facilities, such as washing elephants or touching or hugging their trunks, also cause stress in some individuals [187] and require the same type of harsh training of young elephants to ensure that they can be safely handled (compromising Domains 4 and 5) [173].

Some standards have been established, such as the Asian Captive Elephant Standards (ACES), which aims to improve elephant welfare in Southeast Asia through certification based on husbandry guidelines [188]. However, ACES has failed to safeguard elephant welfare so far because the process is voluntary, and only a few venues have been certified.

The experiences of elephants used for entertainment show examples of welfare compromises across the Domains, resulting in the following negative mental states: hunger, thirst, trauma, discomfort, anxiety, pain, stress, fear, sickness, frustration, boredom, distress, and depression (see Table 5 and Table S10).

## 4. Discussion

The ten case studies in this review provide a snapshot of the welfare compromises encountered in the global commercial wildlife trade. Despite increasing evidence of the capacity of animals (across a range of taxonomic groups) to think, feel, and suffer [17,18,19,20,21,189], our review highlights the profound animal welfare implications experienced by individual animals traded to meet international demand for fashion, pets, luxury food items, and tourism experiences. We infer that billions of animals experience negative mental states, from source to sink, annually.

### 4.1. The Status Quo

Scientific knowledge of how animals think and feel has developed and expanded over recent decades [189], as have the biological and legal principles underpinning animal welfare [190,191]. It is increasingly recognised that all vertebrate species, and potentially all invertebrate species traded globally, are sentient beings capable of experiencing fear, pain, and distress throughout the trade process [17,18,19,20,21,189]. This scientific understanding forms the basis for moral concern regarding their well-being, which is further supported by the recognition of their intrinsic value—the inherent worth an animal holds independently of human interests or societal use [190]. As a result, animal welfare legislation has been established to govern, restrict, and prevent practices that cause unnecessary distress and suffering. However, despite these advances, animal welfare legislation concerning commercial wildlife trade is relatively lacking compared to other key concerns such as sustainability [13]. As a consequence, the intrinsic value and sentience of wild animals are frequently overlooked throughout the commercial wildlife trade chain, leading to the poor welfare of millions of animals [11,14].

Generally speaking, there are two main approaches to wildlife utilisation promoted in science and policy: those that prioritise non-consumptive uses (i.e., that do not involve animals being deliberately bred, killed, removed from the wild, or having their body parts used [14,191]) and those that prioritise regulation of the consumptive use of wildlife [192]. Strategies centred on consumptive use encourage the improved upkeep, control, and expansion of the commercial wildlife trade and work to advance the commodification of wild animals, frequently for purposes of development or conservation [193]. However, approaches prioritising non-consumptive use aim to reduce and eliminate the consumptive commercial use of wild animals in trade and prioritise non-consumptive “animal welfare-friendly” alternatives [33,170,194].

The Intergovernmental Science-Policy Platform on Biodiversity and Ecosystem Services (IPBES) has underscored the complexities of wild species exploitation, highlighting the need for adaptive management and ongoing negotiation [195]. The platform’s recent assessments on sustainable use [195], biodiversity [196], and invasive species [197] emphasise the net negative impact of wildlife exploitation on biodiversity and call for a transformative shift in humanity’s relationship with nature. Notably, the IPBES “Transformative Change” report [198] explicitly acknowledges the need to move beyond a domination-based relationship with nature and embrace relations of care that recognise the agency and sentience of non-human entities, such as animals, plants, and ecosystems [198]. While this is a welcome step, such recognition remains the exception rather than the rule in international policy [13].

Despite the potential of the IPBES framework to influence attitudes, it is not legally binding and faces resistance from existing global agreements rooted in historical approaches. Specifically, the various international bodies, agreements, and legal instruments that have some influence on wildlife trade appear mostly to support or are otherwise interpreted (by governments, businesses, and consumers alike) to prioritise efforts focused on promoting better regulation of the status quo; the goal being legal, sustainable, traceable, safe, and equitable commercial exploitation of wild animals [199]. However, a critical gap remains: these frameworks rarely address the welfare of individual wild animals directly, and sustainable use does not necessarily equate to good welfare for wildlife [14]. On the contrary, sustainable use often perpetuates animal suffering [11,200].

Comparatively, very few international legal instruments appear to support efforts focused on reducing the commodification of all wild animals (regardless of their conservation status), prioritising their non-consumptive use, or acknowledging sentience or the intrinsic value of wildlife [201]. For example, CITES is the main tool for international wildlife trade regulation but is predominantly focused on regulating international trade with respect to sustainable use and trade of CITES-listed species [202]. CITES’s remit is to prevent species threatened by extinction from unsustainable trade, and it offers some protection for the welfare of individual wild animals by requiring the trade to be carried out in a way that “minimises risk of injury, damage to health or cruel treatment” [95]. However, CITES only regulates trade in those species listed in their appendices at a given point in time, and so neglects many taxa (including conservation-threatened taxa) [35], is restricted to international trade (i.e., not domestic), and is dependent on adherence and enforcement by signatory states [104]. As a result, millions of (non-threatened and threatened but not yet listed and regulated under the convention) individual wild animals are not afforded protection [203].

Even for CITES-listed species, Parties to CITES can make reservations and exceptions. For example, CITES regulates the ball python trade but has no remit over the methods used to capture them in the wild or the manner in which they are ranched, stored, or transported [3]. Furthermore, Parties are not currently required to report on animal deaths in transit or at other points in the trade chain, welfare violations, or to re-home or repatriate confiscated animals [59]. Moreover, because CITES regulation applies only to international trade, governments can establish their own national-level laws regarding sourcing, monitoring, and internal trade, even for protected species.

Similarly, another critical international policy forum, the Convention on Biological Diversity (CBD), aims to “preserve and protect nature and its essential services to people” [204,205]. Whilst there is a recognition that Contracting Parties should be “conscious of the intrinsic value of biological diversity” under the CBD’s remit of sustainable use [206], individual animals are not directly considered [201]. The World Organisation for Animal Health’s (WOAH) international standards arguably hold more promise for the well-being of traded animals because welfare criteria have been adopted in its Terrestrial Code [207]. These standards are mostly about how improved animal welfare can help prevent and control zoonotic diseases (e.g., [208]). However, these standards are not legally enforceable, meaning countries are not obliged to prohibit harmful practices [201]. They are also largely oriented towards domestic animals in production systems.

Outside the US and EU, where databases on imported wildlife are maintained (the USFWS LEMIS database and the EU TWIX trade database, respectively), there is very little comprehensive data on which animals are in trade and in what quantities. There have been many suggested approaches to improve regulation and monitoring of the commercial wildlife trade to make the trade more sustainable and equitable. For example, approaches have included the Live Animal Regulations (LARs) from the International Air Transport Association (IATA), certification systems [209], and the use of DNA markers and imaging technologies (e.g., satellite, thermal, and radar) to track wild populations and detect poaching [28]. However, improved regulation of the commercial wildlife trade alone will not necessarily adequately address its negative impacts on animal welfare. Therefore, we posit that there are several key problems with taking the aforementioned type of regulatory action as a default approach that is often ineffective for safeguarding animal welfare rather than efforts to minimise the volume of wild animals traded and prioritise non-consumptive use.

First, greater regulation per se of aspects of the commercial wildlife trade (however well intended) may inadvertently enable animal suffering due to a lack of consideration of sentience and animal welfare needs. For example, the reptile pet trade model depends on systems (using racks for keeping animals) that aim to maximise space and resources. However, rack systems (and regulations stipulating minimum enclosure size, e.g., DEFRA, [210]) fail to recognise aspects of reptile behaviour that are important for their welfare (for example, the importance of snakes to being able to extend their entire body length and to climb and burrow [23,211,212]).

Second, regulations are often based on animals surviving rather than thriving in commercial exploitation [213]. For example, stricter regulations on housing wild animals in captivity can result in marginal improvements. However, such regulations fail to address the fact that wild animals (such as dolphins used for circus-like performance experiences) may suffer decades of stress and physical pain simply being in captivity, which improvements can do little to alleviate.

Third, the trade may depend on practices widely considered as being inherently inhumane and where its prohibition is the only way to protect animal welfare; for example, in the case of the harsh training methods used to ensure that captive elephants can be safely handled and controlled for use in interactive tourism [173,180].

Fourth, regulatory measures tend to be restricted to part of the wildlife trade chain. For example, the IATA LARs only come into force when wild animals are loaded onto an airline. As a result, in the absence of national laws (in source and end-use countries), animal welfare issues and (pre-export) mortalities during capture, confinement, land transport, farming, and captivity are typically ignored (e.g., see [1]).

Lastly, regulatory measures may be difficult to implement and monitor. For example, some commercial captive lion breeding facilities in South Africa use legal activities, like captive breeding and canned hunting, to fuel (and potentially cover) their involvement in the illegal international big cat bone trade [214]. Some of these facilities also facilitate inhumane hunting practices that are non-compliant with provincial regulations pertaining to the industry [215]. In addition, in West Africa, it has been reported that wild animals such as tortoises may be packaged for shipping for international commercial trade in such restricted spaces that they are highly unlikely to be able to fully extend their head and neck during the journey [3], potentially in violation of the IATA LARs [3].

These examples are not exhaustive but illustrate the potential shortcomings of seeking to improve regulatory action alone. In summary, whilst current regulatory efforts are aimed at making the trade more sustainable and equitable, the sheer scale, breadth, and complexity of the international commercial wildlife trade frequently imply that meaningful regulatory action is challenging to enact, and measures often only lead to marginal improvements that do little to alleviate the negative impacts on animal welfare.

### 4.2. Time for a New Approach?

If we cannot prevent wild animals from suffering as a result of their commodification nor provide them with what they need and prefer, then arguably, a more systemic change in mindset is necessary. To this end, a significant shift in demand toward non-consumptive uses of wildlife is required. This shift would involve reducing the commercial consumptive exploitation of wild animals and redirecting demand toward “wildlife-friendly” alternatives. For example, tourism activities could focus on ethically managed, hands-off interactions with wild animals in their natural environments, such as wildlife parks, whale watching, or photo tourism [180]. Similarly, synthetic substitutes or responsibly sourced herbal alternatives could replace traditional medicines that rely on ingredients derived from wild animals [216].

To achieve such change, lessons can be drawn from frameworks in other fields, such as the 3Rs framework, which was originally developed in relation to animal research. The 3Rs seek to replace and reduce the use of sentient animals and to refine procedures to reduce suffering [217]. The 3Rs can be problematic as they set only minimum standards, which are not always adhered to. However, the concept may still serve as a foundation for developing a new approach. A similar framework could be applied to the commercial wildlife trade to mitigate harm and significantly improve animal welfare. This is particularly important given the availability of numerous “wildlife-friendly” alternatives that can replace consumptive practices. Relatedly, current welfare assessment models used as part of regulatory frameworks are under-applied. Future applications would also benefit from the requirement of the precautionary principle of assigning the benefit of the doubt to animals [218]. Relevant criteria should aim to promote approaches prioritising animal-centric preferred life quality to guide health and welfare controls [22].

In light of the overwhelming evidence of animal suffering in trade [11], the recognition of their sentience and intrinsic value [17,18,19,20,21,189], growing public concern about animal welfare [219,220], and the expanding scope and intensity of the wildlife trade [6], there is an urgent need to adopt this type of transformative approach. Consequently, while improvements in welfare regulation through mechanisms such as CITES and WOAH should be welcomed in the short term, we posit that these measures alone cannot address the fundamental issues inherent to the wildlife trade.

Moving to a transformative approach, reducing and redirecting demand towards non-consumptive use, which does not exploit, kill, or take animals from the wild, has wider benefits beyond animal welfare. For example, from a conservation perspective, species threatened with extinction due partly to trade, but for which such data and evidence are lacking [6], could benefit greatly. Public health may also benefit because the exploitation of wildlife has been identified as one of the dominant drivers of zoonotic disease transmission [221,222,223]. The scale of international wildlife trade facilitates and exacerbates the spread of diseases on a global level, potentially leading to pandemics and severe negative impacts on people and economies [224,225,226]. Ensuring better welfare standards within the trade could help mitigate these risks, as poor welfare conditions often exacerbate stress, injury, and disease transmission among animals, increasing the likelihood of spillover events [222]. Furthermore, whilst the argument for maintaining commercial trade is often based on economic benefits for local communities [105,164,227], over-reliance on wildlife trade can be precarious, as unsustainable practices threaten long-term resource availability and the economic stability of developing countries [203]. Many species targeted in trade are already declining, meaning communities risk losing a key source of income over time [24,203]. Wildlife trade also risks perpetuating inequality because global trends show that commerce typically flows from poorer exporter nations to wealthier importers [222]. Local communities and those living close to wildlife often benefit the least when involved in trade activities [228] and suffer the slowest economic recovery following zoonotic disease outbreaks [229]. As seen in case studies Section 3.4 and Section 3.5, demand for wildlife products may be driven by cultural and medicinal beliefs. While these traditions remain significant, shifting societal attitudes and increasing availability of synthetic or sustainable alternatives present opportunities to reduce reliance on wildlife use without disregarding cultural heritage [33,71,103,216]. Promoting higher welfare standards and transitioning towards alternative livelihoods, such as ecotourism, community-based conservation programmes, sustainable agriculture, and the development of plant-based or synthetic medicinal alternatives, could provide more stable and ethical income sources while reducing dependence on wildlife exploitation [230,231,232]. Lastly, the introduction of non-native species through legal and illegal trade poses significant ecological and economic risks, further highlighting the need for stricter regulation and demand reduction [233,234].

## 5. Conclusions

The case studies show how individual sentient animals involved in the wildlife trade typically experience severe welfare compromises across the Five Domains, including (1) poor nutrition through often a severe deprivation of food and water or inappropriate and insufficient provisions; (2) a poor environment, both on a short and long-term basis, because animals are often exposed to severe restriction and confinement, thermal extremes, and an unpredictable environment; (3) poor health, because many animals experience ill-heath through the transmission of diseases, poor hygiene, compromised immunity, injuries, as well as prolonged and typically inhumane deaths; and (4) poor behaviour because individual animals are unable to exercise control or agency over their lives, are prevented from performing important and natural behaviours, are kept isolated or in inappropriate social groupings, and experience negative interactions with humans. The compromises seen across these four domains can all result in an overall negative mental state for these animals, with feelings of pain, stress, fear, discomfort, frustration, depression, boredom, hunger, thirst, and distress being likely mental states (Domain 5) experienced by traded wild animals at specific or regular points in their trade journey.

In light of the accelerating biodiversity crises, the widespread and devastating effects of zoonotic diseases, and the increasing knowledge and understanding of the sentience of animals and what this means for how we treat them, it is important that we seek to shift away from commercial consumptive practices and urgently prioritise non-consumptive approaches that respect the sentience, intrinsic value and welfare of all animal species.

## Figures and Tables

**Table 3:** The experiences of crickets (*Gryllidae*) and frogs (*Anura*) in the commercial wildlife trade for luxury meat, presented in three metrics; the estimated numbers of animals involved, the potential duration of their experiences for each phase of the trade, and the severity of their welfare compromises, described using the Five Domains model. NA: Not applicable.

				Severity of Welfare Compromises				
Species	Numbers	Trade Stage	Duration	Nutrition	Environment	Health	Behaviour	Mental States
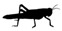	~370 billion—430 billion slaughtered or sold live or dead/yr [119].	**Rearing**	Up to 2 months (longer for breeders)	Inappropriate food	Overcrowding; barren and restricted environment, space and complexity	Risk of disease, injury and cannibalism from overcrowding; risk of mortality from misguided or poor husbandry	Significant constraints on behaviour; lack of control	Discomfort, pain, stress, fear, sickness, boredom, and frustration
		**Slaughter**	Seconds to hours	Feed withdrawal	N/A	Inhumane slaughter methods	N/A	Hunger, fear, and pain
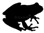	~100–400 million/yr internationally ~814 million to 2 billion were imported into the EU between 2011 and 2020 [130].	**Capture and Transport**	Hours to days	Restricted water and food availability	Thermal extremes likely; absence of damp substrate and water; close confinement; crowded conditions; unpredictable events/noises	Risk of disease and injury from capture and close confinement; Risk of suffocation and crushing from holding method	Barren and inappropriate environment; lack of control; significant constraints on behaviour; negative interactions with humans	Thirst, hunger, discomfort, pain, stress, fear, sickness, exhaustion, and distress
		**Market**	Hours to days	Restricted water and food availability	Thermal extremes likely; absence of damp substrate and water; close confinement; crowded conditions; unpredictable events/noises	Risk of disease and injury from capture and close confinement; Risk of suffocation and crushing from holding method	barren and inappropriate environment; lack of control; significant constraints on behaviour; negative interactions with humans	Thirst, hunger, discomfort, pain, stress, fear, frustration, sickness, exhaustion, and distress
		**Slaughter**	Seconds to hours	N/A	N/A	Inhumane, slow and painful slaughter	Negative interactions with humans	Pain, fear, discomfort, distress, and stress

**Table 5:** The experiences of lions (*Panthera leo*) and elephants (*Elephas maximus*) in the commercial wildlife trade for animals in entertainment, presented in three metrics; the estimated numbers of animals involved, the potential duration of their experiences for each phase of the trade, and the severity of their welfare compromises, described using the Five Domains model.

				Severity of Welfare Compromises				
Species	Numbers	Trade Stage	Duration	Nutrition	Environment	Health	Behaviour	Mental States
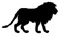	~8500 in almost 400 facilities in South Africa [163,164].	**Farming**	Years	Early weaning; inadequate and insufficient diets; dirty water	Small and overcrowded housing; barren environments; exposure to high temperatures during forced walks	Malnourishment; painful injuries and fractures from handling; low reproductivity; high cub mortality; poor immune functioning; inbreeding; lack of proper veterinary care; increased risk of disease outbreaks	Inappropriate and crowded groupings; bullying between lions; early weaning/separation; negative and forced interactions with humans for petting and walks; repeated separation from other cubs; inhumane training practices	Hunger, thirst, trauma, discomfort, anxiety, pain, stress, fear, sickness, frustration, distress, boredom, and depression
		**Killing**	Minutes to hours	If canned hunting may be feed- and water-restricted	If canned hunting then kept in isolated confinement	Pain resulting from inhumane killing practices, including canned hunting and killing for parts	Negative interactions with humans; isolation	Hunger, thirst, trauma, discomfort, anxiety, pain, stress, fear, sickness, frustration, and distress
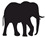	~3800 in elephant camps across Southeast Asia [173]	**Phajaan**	Weeks	Starvation; inappropriate and varied diet; water deprivation	Severe restriction and confinement	Pain and injury; risk of disease and mortality	Highly restricted behaviour and confinement; stereotypies; inhumane training for performance of unnatural tricks; traumatic experiences; PTSD	Hunger, thirst, trauma, discomfort, anxiety, pain, stress, fear, sickness, frustration, boredom, distress, and depression
		**Captivity**	Years	Inappropriate and unvaried diet; water deprivation at night	Severe restriction and confinement	Pain and injury; risk of disease and mortality	Highly restricted behaviour and confinement; stereotypies; inhumane training for performance of unnatural tricks; traumatic experiences; PTSD	Hunger, thirst, trauma, discomfort, anxiety pain, stress, fear, sickness, frustration, boredom, distress, and depression

## Supplementary Materials

The following supporting information can be downloaded at: https://www.mdpi.com/article/10.3390/ani15070971/s1, Table S1: Ball pythons (*Python regius*) for the pet trade. Table S2: Zebrafish (*Danio rerio*) farmed for the pet trade; Table S3: African Grey parrots (*Psittacus erithacusor*) for the pet trade; Table S4: Sharks for the fin trade; Table S5: Pangolins for traditional medicine; Table S6: Crickets (*Gryllidae*) for feed and food; Table S7: Frogs (*Anura*) for the frog-leg trade; Table S8: Crocodilians (Crocodylidae) farmed and killed for their skins; Table S9: Lions (*Panthera leo*) on commercial farms; Table S10: Elephants (*Elephas maximus*) used for tourist rides. References [17,235,236,237,238,239,240,241,242,243,244,245,246,247,248,249,250,251,252,253,254,255,256,257,258,259,260,261,262,263,264,265,266,267,268,269,270,271,272,273,274,275,276,277,278,279,280,281,282,283,284,285,286,287,288,289,290,291,292,293,294,295,296,297,298,299] are in the Supplementary Materials only.
