# Supplementary material for: Chains of Commerce: A Comprehensive Review of Animal Welfare Impacts in the International Wildlife Trade
Source: Animals (Basel). 2025 Mar 27;15(7):971. doi: 10.3390/ani15070971 (PMC11988014; doi:10.3390/ani15070971)
Supplement: Supplementary file 1 [file animals-15-00971-s001.zip › Table S10_elephants.pdf]

Table S10: Elephants (*Elephas maximus*) used for tourist rides

Detailed explanation for the welfare compromises described in Table 5 for the trade of elephants for entertainment.

**Elephants used for tourist rides**
**Numbers:**<br>Across Southeast Asia, there are around 3800 captive elephants kept in camps for tourists to ride, interact with, or observe [175]. The vast majority of them are captured from the wild, and despite common misconceptions, are not domesticated animals [177]. In recent years, following the ban on logging in Thailand in 1989, the use of elephants in Thailand, and elsewhere in Asia, became more intensive [187]. As a result, the well-being of these long-living animals has worsened significantly [187]. The monetary value of elephants has also risen sharply, and far outweighs the fine for poaching a wild elephant, which does little to discourage the practice [292]. What was once a traditional practice, where mahouts had long apprenticeships, and 'inherited' the career and the elephants from previous generations, is now seen as an undesirable job, and as a result, the welfare of both mahouts and elephants has suffered greatly [187]. For example, the conditions in which elephants are kept have typically worsened, and elephants are given fewer freedoms than when used by traditional mahouts [187].
**Duration of experiences:**<br>Elephant calves are taken from their mothers at an early age (1-2 years), and 'broken' (*phajaan)* at the age of 3 or 4 yrs old and kept until they can no longer work [83]. Elephants may live for 70 years in the wild, but this is typically shorter in captivity [293].
**Severity (welfare compromise using the Five Domains Model):**
1. Nutrition<br>- Starvation is likely during *phajaan*<br>- Inappropriate and unvaried diet<br>- Water may be withdrawn during *phajaan*<br>- Water is usually limited or not available at night
Evidence for Nutrition welfare compromises<br>Phajaan is likely to involve periods of starvation and water deprivation, which could be for days or even weeks [294].

Diet is limited and unvaried and may be overly dependent on sugary foods, such as sugar cane and bananas, which can result in health issues [177,295]. Food quantities may be insufficient, resulting in malnutrition and chronic hunger [6].

Water is typically not provided at night, and is not given in sufficient quantities during the day, and the effects of this may be exacerbated by high environmental temperatures and a lack of shade [6].

2. Environment
- Severe restriction and confinement during *phajaan*
- Severe restriction and confinement, either by pen or chains, for the remainder of their life

Evidence for Environment welfare compromises

Elephant calves and older elephants are confined and restricted for their entire working lives, whether in enclosures or by chains, other than for short periods for tourist interactions [294]. The poor environmental conditions that captive elephants used in this way experience contributes to the development of long-term emotional and behavioural pathologies [296].

Long-term captivity is known to be a considerable stressor for elephants, and this is exacerbated by the extreme degree of confinement seen in these elephants [297].

3. Health
- Pain and injury from practices during *phajaan,* from training and control methods, and the use of chains
- Risk of disease and mortality from improper husbandry practices

Evidence for Health welfare compromises

Injuries can cause significant pain and suffering in elephants and can be repetitive [298]. These injuries result from improper housing and confinement, and from the inhumane training and control methods that can be used. This continuous infliction of pain and suffering is known to be a contributory factor to the development of psychological illnesses such as post-traumatic stress disorder (PTSD) in elephants [297].

Disease and premature mortalities are common in tourist camps and may cause extensive pain and suffering to the elephants, especially when veterinary treatment is limited or unavailable [296].

Isolated housing is known to contribute to psychological and physiological stress, and be a factor in early mortality rates [299].

4. Behaviour

- Highly restricted behaviour, as removed from natural social grouping and mother, kept confined or chained, and trained to perform unnatural tricks and behaviours, often using inhumane training methods.<br>- The trauma experienced is known to result in post-traumatic stress disorder in rescued elephants.
<u>Evidence for Behaviour welfare compromises</u><br>Severe confinement and prevention from natural behaviours are commonly seen in elephants kept at tourist camps and are known to result in significant stress, and even chronic mental health disorders such as PTSD [297]. Isolated housing and social deprivation are known to contribute to psychological and physiological stress and are a factor in early mortality rates [299]. The elephants are also typically unable to form bonds with other elephants, which can have considerable impacts on their welfare, as elephants use peers as social supports, and to buffer stressful experiences [186].
5. <u>Mental State</u>: Potential affects arising from domains 1-4 include;<br>➢ (1) Hunger and thirst<br>➢ (2) Discomfort, pain, stress, and fear<br>➢ (3) Sickness, pain, discomfort, fear, and stress
<u>Mental state welfare compromises</u><br>Elephants are complex, intelligent and sentient animals, and the welfare compromises in the previous four domains can give rise to a range of potential affects, and given the particularly long duration of these welfare compromises, the severity of these affects may be considerable.
