# Supplementary material for: Chains of Commerce: A Comprehensive Review of Animal Welfare Impacts in the International Wildlife Trade
Source: Animals (Basel). 2025 Mar 27;15(7):971. doi: 10.3390/ani15070971 (PMC11988014; doi:10.3390/ani15070971)
Supplement: Supplementary file 1 [file animals-15-00971-s001.zip › Table S1_Ball pythons.pdf]

Table S1: Ball pythons (*Python regius*) for the pet trade
Detailed explanation of the welfare compromises described in Table 1 for the trade of ball pythons for exotic pets.

Ball pythons for the pet trade

Numbers:
~100,000 exported annually, including from 'ranches' or 'captive-breeding facilities [39,238].

Ball pythons (*Python regius*) are one of the world's most highly traded reptiles and the most traded African species listed on CITES [39,239]. Around 100,000 ball pythons are legally exported annually under CITES permits from West Africa, primarily to meet high international demand in the USA, Asia, and Europe for pets [39,238]. Besides international demand for the pet trade, West Africa also has a high domestic demand for ball pythons for use as traditional medicine and bushmeat [240]. Most ball pythons exported internationally as pets are ranched, a process that depends on the supply of eggs, neonates, and juvenile specimens captured from the wild [241]. In addition, captive breeding of ball pythons for specific colours and patterns is increasing due to the growing desire for rare or unique-looking pets [34,39,42]. Although, there are concerns regarding the misreporting of CITES source codes for 'reportedly' captive-bred individuals [39,40,42].

Duration of experiences:
Ball pythons are captured and transported to holding facilities before being sold at markets, moved to farms or ranches, or transported internationally for the pet trade.

<u>Capture</u>: Hours to days
- Opportunistic captures
- Digging out of burrows/tree trunks
- Eggs, juveniles or adults may be wild-caught
- Held in sacks/ confined for varying periods

<u>Transportation</u>: Hours to weeks
- Transported via sack or small container at multiple points in the trade chain.

<u>Exotic pet trade</u> (pet shops, exhibitions, online traders): Weeks to years
- Ball pythons may be exhibited by traders, either in shops, online, or in exhibitions, typically whilst confined in a rack system.
- Duration is dependent on how long it takes for the individual to be sold.

Exotic pet ownership: Weeks to years

- Dependent on several factors, including the survival of the individual and the commitment of the owner, who may relinquish the snake back into the trade.

Severity (welfare compromise using the Five Domains Model):

1. Nutrition

- Restricted water and food intake (capture and transportation)
- Food is likely to be nutritionally adequate, although presented in a way that removes their ability to perform natural hunting behaviours (exotic pet trade and ownership)
- Water containers are commonly too small compared with what is recommended (exotic pet trade and ownership)

Evidence for Nutrition welfare compromises

During capture and transportation, Ball pythons may not have access to water or feed for hours, days or weeks. Ball pythons do have a degree of resistance to periods of food and water withdrawal, though, as they can last for over a week without feeding and drinking [242, 243]Click or tap here to enter text.. However, juvenile ball pythons should be fed weekly, and a lack of water to drink when required has a degree of welfare compromise, especially when experienced for a longer period [242,243].

Malnutrition is a concern for pets and exhibited snakes, particularly as they may refuse to feed due to stress [215,244].

Food is typically presented in unnatural ways due to the ethical concerns associated with feeding live prey [244].

Water containers seen in pet exhibitions are typically too small compared with what is recommended [34,245].

2. Environment

- Thermal extremes likely (capture, transportation and exotic pet trade)
- Close confinement with an absence of light and fresh air (capture, transportation, and exotic pet trade)
- Unpredictable events/ noises are likely when held in a sack and carried around, as well as when transported, exhibited for trade, and kept as exotic pets (all phases)
- Lack of enrichment, substrate, and water to bathe in (all phases)
- Barren environment (all phases)

Evidence for Environment welfare compromises

Although ball pythons live in countries with temperatures ranging between 16°C – 43°C, they perform behavioural thermoregulation to cope with the temperature and humidity conditions, including seeking out heat spots and cool burrows depending on their needs. These behaviours are prevented when held in a sack, and high temperatures may also be exacerbated by being held with other snakes in crowded conditions.

Typically, during capture and transportation, ball pythons are held in bags for some time and may also be transported in these bags or transferred to equally small containers, including small boxes [40,43].

Transportation and placement in multiple places for exhibiting can result in various thermal conditions, especially when catering for thousands of species in one location [34]. Reptiles have specialist needs, and one species will have different needs from another, although both are kept in the same rack system [34,216].

Transportation, pet exhibitions, and life as a pet are all likely to involve a range of unpredictable noises and events, which are known to be stressful for captive animals [62].

It is unclear whether ball pythons need water to bathe in or whether their welfare suffers without it. Given the fact that other snake species do require this, it may be that its omission results in welfare compromises [34].

D'Cruze et al. [34] assessed the housing conditions of 4855 ball pythons in six different pet expositions and a further 787 ball pythons from YouTube videos. All the snakes in the pet expositions were kept in enclosures smaller than their length, preventing them from fully stretching out, often missing shelters and water, and most had either none or inadequate substrate. None of the vendors provided any enrichment for the snakes, nor did they provide any information about how to care for them properly. Similar issues were found for the snakes featured on YouTube, as almost all of them were kept in enclosures smaller than their body length, and most did not have sufficient access to water, shelter, or enrichment [34].

The common use of display and rack systems used by breeders, pet shops, and exhibitors considerably limits the mobility of snakes, as the enclosures prevent them from adopting straight line body postures and moving properly in any direction. Although ball pythons are considered to be sedentary, there has been little consideration for the impact that such restricted movement has on their wellbeing, and even sedentary snakes need to straighten their body to alleviate discomfort, avoid stress fully, and exercise [23,215,245,246].

The rack systems also do not allow space for shelters, which, for reclusive animals like ball pythons, are likely to be important features in their environment [34,214,243,245]. The length of time that ball pythons will spend in these small, barren enclosures typically varies, and exhibitors will argue that they are temporary housing facilities [247]. However, the rack systems seen for housing ball pythons on YouTube suggest that these snakes are kept in these systems permanently, which could easily result in them spending weeks, months or even years in these highly restricted conditions [34].

Ball pythons and other captive reptiles are known to perform stereotypical and abnormal behaviours in response to captivity, particularly when highly confined and kept in a barren environment [48,214]. Confinement is known to be stressful for ball pythons, and captive ball pythons show increased plasma corticosterone levels following being handled, held in a moving sack for one hour, and then placed into a small container, compared with their baseline levels [46]. Unpredictable events and noises are known sources of stress for animals in captivity, especially when other senses, such as sight, are unavailable [248]. The pythons may be kept in these conditions for sale for days, weeks, months or even years at a time.
Stereotypic interactions with enclosure walls, which are the result of captivity stress, are often ignored by owners [48,214]
.
Rapid open-mouth breathing may be seen as 'normal' by owners, yet it is associated with hyperthermia due to inappropriate thermal conditions [249].

3. <u>Health</u>

- Risk of disease, injury and suffocation from close confinement and crowded housing with other animals (capture and transportation)
- Evidence of high morbidity rates in breeding and holding facilities (capture and transportation)
- Physiological stress from intense confinement may be responsible for high mortality rates (all phases)
- Potential for ill health when not kept under correct husbandry protocols, which are complex and not always fully understood by pet owners (exotic pet trade and ownership)

<u>Evidence for Health welfare compromises</u>

The capture process poses a risk of injury and increased susceptibility to disease, infection and eventual mortality, especially when different species are confined in close proximity [40,250].

Diseases and injuries are a common concern for wild-caught and ranched ball pythons and are exacerbated by close confinement and overcrowding and their associated physiological stressors [40,42,43]. The impact of these is a high mortality rate in ball pythons, which has been reported as being as high as 33% during transportation [43,44].

Visits to "holding facilities" in West Africa have also revealed sub-optimal welfare conditions, with morbid animals receiving no veterinary care and no routine hygiene or disease transmission protocols in place [40].

Little research has been performed into the effects of physiological stress in all pythons, but this could be a reason for the high mortality rates in these animals [34].

Snakes need to be able to exercise, move, and stretch out fully to have good health, but the severe confinement of rack systems prevents this [34,245].

Due to their specialist needs, ball pythons are subject to many potential welfare concerns, resulting in mortalities of pet reptiles in their first year ranging from 3.6% - to 75% [251,252].

There are often challenges for owners to find appropriate specialist veterinary guidance, exacerbated by the lack of information provided at the point of sale [34,216].

4. Behaviour

- Barren and inappropriate environment, no freedom to make choices, and significant constraints on behaviour for long periods, including being unable to unfurl or straighten into full length (capture, transportation exotic pet trade, and potentially also in exotic pet ownership)
- Negative interactions with humans (potentially all phases)

Evidence for Behaviour welfare compromises

The hunting process is likely to be stressful for the snakes, as they are wild animals who are preyed upon by a range of terrestrial and aerial predators and are not used to being handled by humans [46].

Their limited housing significantly restricts the behaviour of the ball pythons, and they cannot stretch out fully, move, feed, drink, or perform behavioural thermoregulation. Studies have shown that confinement, whether in a bag or on a rack system, prevents important behaviours in ball pythons, significantly impairing their welfare and resulting in physiological and psychological stress [46,214]. Furthermore, snakes quickly lose muscle tone and fitness when restrained from movements, which can have long-term negative effects [22].

The common use of display and rack systems that are used by breeders, pet shops, and exhibitors considerably limits the mobility of snakes, as the enclosures prevent them from adopting straight-line body postures, and from moving properly in any direction. Although ball pythons are considered to be sedentary, there has been little consideration for the impact that such restricted movement has on their wellbeing, and even sedentary snakes need to straighten their body to alleviate discomfort, avoid stress fully, and exercise [23,215,245,246].

Snakes can also find handling stressful, resulting in negative interactions with humans, especially for wild snakes used to aerial and terrestrial predators [46].

5. Mental State: Potential affects arising from domains 1-4 include;

- ➢ (1) Thirst and hunger
- ➢ (2) Discomfort, pain, stress, and fear
- ➢ (3) Sickness, pain, discomfort, fear, and stress
- ➢ (4) Exhaustion, fear, pain, and distress

Mental state welfare compromises<br>Welfare compromises in the previous four domains have the potential to give rise to a range of affects that snakes, as sentient beings, are known to be capable of experiencing [20].
