# Supplementary material for: Chains of Commerce: A Comprehensive Review of Animal Welfare Impacts in the International Wildlife Trade
Source: Animals (Basel). 2025 Mar 27;15(7):971. doi: 10.3390/ani15070971 (PMC11988014; doi:10.3390/ani15070971)
Supplement: Supplementary file 1 [file animals-15-00971-s001.zip › Table S2_Zebrafish.pdf]

Table S2: Zebrafish (*Danio rerio*) farmed for the pet trade
Detailed explanation of the welfare compromises described in Table 1 for the trade of captive-bred zebrafish for exotic pets.

Zebrafish farmed for the pet trade

Numbers:
Unknown, but over 5 million are thought to be bred for research each year [83].

Although most fish in the wildlife trade are destined for consumption, many are caught and captive-bred for the rapidly growing pet trade [65,253]. Accurate figures regarding the scale of the trade in pet fish remain unclear as so much of it is unmonitored, and few scientific analyses have attempted to quantify the numbers involved [65]. The global trade in ornamental fish was valued at USD 5.4 billion in 2021 and is estimated to grow 8.5% annually up to 2030 [254]. Approximately 90% of ornamental freshwater fish species destined for pet shops are captive-bred, compared with around 5-10% of ornamental marine fish [255]. Zebrafish are typically captive-bred, and the number of genetic lines now exceeds 44,000 [256]. As well as a popular pet species, zebrafish are also used extensively in research, and over 5 million are thought to be used in laboratories worldwide each year [52].

Duration of experiences:
Captive breeding: Days to weeks, or 2-3 years for breeders [253].
Zebrafish are captive-bred in different systems and containers, are routinely graded and re-housed, and are subject to continual regrouping before being exported.

Transportation: Hours to days
- Transported typically by water and air-filled bags.

Exotic pet trade (pet shops, exhibitions, online traders): Weeks to years
- Zebrafish may be exhibited by traders, sold in pet shops or sold online. Typically kept in small and often barren tanks.
- Duration depends on how long the individual takes to be sold.

Exotic pet ownership: Weeks to years
- Dependent on several factors, including the survival of the individual and the commitment of the owner, who may relinquish the fish back into the trade.

Severity (welfare compromise using the Five Domains Model):

1. Nutrition

- Potential for food to be restricted during transportation and for 24hrs before transport (transportation)
- Food is otherwise considered to be acceptable nutritionally, but pellet/flake feeding removes the natural feeding behaviour and control over feeding (captive breeding, exotic pet trade, and exotic pet ownership)

Evidence for Nutrition welfare compromises

Feed is likely to be withdrawn for transportation, which is considered to be acceptable on welfare grounds for 2-3 days [257]. Zebrafish may also be starved for 24 hours before transportation to reduce waste production and maintain water quality [257]. There is potential for the impact to be very low, particularly as there are welfare impacts from feed withdrawal in terms of their experiences during transportation. However, too long a period of starvation can increase aggression, stress and injuries [62].

In the wild, zebrafish consume various foods, including insects, arachnids, and detritus from both the surface and round level, and will feed throughout the day [258].

2. Environment

- Continual changes in the captive environment over the rearing period, and then when in trade until their endpoint (captive breeding, exotic pet trade)
- Severe confinement during transportation (transportation)
- Poor water quality/ oxygen content can have a significant impact on fish welfare and is specifically at risk during transportation and in poorly managed tanks (all phases)
- Thermal extremes are possible during transportation (transportation).
- Unpredictable events/ noises (all phases)
- Barren tanks result in boredom and welfare issues, including stereotypic behaviour (captive breeding, exotic pet trade, and exotic pet ownership)

Evidence for Environment welfare compromises

During captive breeding, fish are typically housed in a series of containers, increasing in size as they grow. It is unclear what effect these changes have on the welfare of the fish.

Overcrowding is a stressor for fish that negatively impacts their welfare [259,260]. Zebrafish are often kept in crowded tanks, with limited space per fish available to them, as well as overall being limited in space. Overcrowding can also degrade water quality and reduce the amount of dissolved oxygen available for the fish, which can impede their welfare and result in more frequent water changes [10].

Water changes are stressors for zebrafish, resulting in temporarily increased stress levels and decreased affiliative behaviours [57].
Transportation may result in their environmental space being even more restricted, potentially reducing water quality [257].
Thermal extremes are possible during transportation, although possible to manage and maintain [257].
Unpredictable events and noises are known to be stressful to fish and are potential risks during captive breeding and transportation [62].

We searched the internet for examples of ornamental fish breeders for the pet industry and found numerous images of overcrowded and barren tanks like those commonly seen in pet shops.

Zebrafish will perform stereotypical behaviours when kept in barren tanks [55, 56]. Captive fish show reduced stereotypical behaviours and signs of boredom when provided with forms of enrichment [261–263].

Zebrafish are known to have increased morbidity and mortality when exposed to husbandry stress, including poor water quality [58].

3. <u>Health</u>

- Risk of disease from close confinement and crowded housing
- Risk of painful injuries from netting and aggressive individuals
- Risk of poor health from mismanaged water quality
- Risk of transportation stress and associated morbidity and mortality
- Risk of husbandry stress from husbandry practices, including water changes

<u>Evidence for Health welfare compromises</u>

There is still a widespread attitude that fish are disposable and that a certain percentage of mortalities are expected [53,255].

Diseases are a significant threat to the welfare of captive-bred ornamental fish, such as zebrafish, and viral outbreaks on large-scale farms have been reported to wipe out whole facilities of fish [62]. The risk is particularly exacerbated by overcrowding, common in breeding facilities, and even more considerable during transportation [62,257,259,260].
Injuries are also a potential welfare concern and can arise at various points, including capture and handling, grading, and aggressive attacks from other fish [53]. Viral outbreaks can have devastating impacts on disease and mortality rates, particularly in large-scale facilities [62].

Water quality is essential for optimum health and welfare, and imbalances can easily occur, especially in overcrowded tanks or transportation. The effects of this can be long-term [53,62]. For example, zebrafish have increased morbidity and mortality when exposed to husbandry stress, including poor water quality [58].

Transportation stress may result in mortality during the journey or potentially following the journey, as the effects are not always temporary or short-term [62]. Stress levels can rise significantly during transportation and remain elevated over longer journeys, suggesting that fish do not acclimate [61]. Transport regulations often allow fish to be transported far longer than they can physiologically cope with [62]. If the transport-related stress is too significant, fish may not recover, resulting in long-term welfare concerns and mortality [62]. If managed well, fish can be transported without apparent physiological impact, although little research has been performed into the long-term effects and the psychological impact of transportation.

Mutilations in ornamental fish are another concern for the trade, and dye-tattooed or dye-injected fish are sold globally [264]. Concerns associated with these often invasive procedures include unnecessary pain and suffering, increased mortality rates, changes in behaviour, increased prevalence of kidney failure and skin disease, and increased vulnerability to aggressive attacks from other fish [264].

4. Behaviour

- Barren and restricted environment, no freedom to make choices, and significant constraints on behaviour (all phases)

Evidence for Behaviour welfare compromises

Fish respond by showing an improved range of behaviour, reduced stereotypies and aggression, and improved stress levels when kept in enriched environments, which suggests that barren tanks negatively impact their welfare [261–263]. Fish may be kept in barren environments for the entire phase of trade and, therefore, experience considerable restriction in behaviour.

It is unclear whether captive-bred fish still maintain their wild instincts and find the severe behavioural restrictions inherently stressful. Some suggest that this may be the reason for unexplained mortalities or high morbidity rates in ornamental fish [53].

5. Mental State: Potential affects arising from domains 1-4 include;

- (1) Hunger
- (2) Discomfort, pain, stress, and fear
- (3) Sickness, pain, discomfort, fear, frustration, and stress
- (4) Exhaustion, fear, frustration, pain, and distress

Mental state welfare compromises

Welfare compromises in the previous four domains have the potential to give rise to a range of affects that fish, as sentient beings, are known to be capable of experiencing [18].
