# Supplementary material for: Chains of Commerce: A Comprehensive Review of Animal Welfare Impacts in the International Wildlife Trade
Source: Animals (Basel). 2025 Mar 27;15(7):971. doi: 10.3390/ani15070971 (PMC11988014; doi:10.3390/ani15070971)
Supplement: Supplementary file 1 [file animals-15-00971-s001.zip › Table S3_African grey parrots.pdf]

Table S3: African Grey parrots (*Psittacus erithacus*) for the pet trade

Detailed explanation of the welfare compromises described in Table1 for the wild-capture and trade of African Grey Parrots for the pet trade.

African Grey parrots for the pet trade

Numbers: African grey parrots (*Psittacus erithacus*) are commonly traded for food, medicine and the international pet trade, the latter being the most significant regarding the number of animals involved [70,71]. In 2016, when Grey parrots were listed in CITES Appendix II, over 1.3 million wild-caught Grey parrots had been exported from 18 range States since 1975 [59,72]. This makes the Grey parrots the most traded of all CITES-listed birds, representing 11% of all reported parrots in the wildlife trade [59,72]. Even since Grey parrots were listed in Appendix I of CITES in 2017, illegal trafficking continues, with numbers unknown [74].

Duration of experiences:

Capture: Minutes to days

- Taking chicks from nest cavities [76].
- Glue and stick method – poles covered in sap used to bind roosting birds' wings together, or sticky substances on branches at feed sites [75]
- Fishing nets – either on the floor at feeding sites or roosting sites where the birds are chased into them [75].

Transportation: Days to Weeks

- Transported in bags to holding facilities [265]
- Holding facilities are overcrowded, with no perches and little to no food and water [72]
- Due to the illegal nature of the trade, the parrots may be concealed in exports of other parrot species [74]

Exotic pet trade (pet shops, exhibitions, online traders): Weeks to years

- Parrots may be exhibited by traders, either in shops, online, or in exhibitions, typically whilst confined in a small cage
- Duration is dependent on how long it takes for the individual to be sold

Exotic pet ownership: Weeks to years

- Dependent on several factors, including the survival of the individual and the commitment of the owner, who may relinquish the snake back into the trade.

Severity (welfare compromise using the Five Domains Model):

1. Nutrition
   - Restricted water and food intake, potentially for weeks (capture and transportation)
   - Food is likely to be nutritionally adequate, although presented in a way that removes their ability to perform natural foraging behaviours (exotic pet trade and ownership)

Evidence for Nutrition welfare compromises

Mortality rates during capture and transportation can be high due to a lack of food and water (among other factors [71,75]. Analyses of social-media listings of African Grey parrots have found that basic animal welfare standards are frequently breached during transportation and in holding facilities, with parrots being given infrequent or no food and water [72]. Feed and water restriction for long periods is highly damaging for birds and can result in extensive suffering and mortalities [250]. These welfare issues may be further exacerbated in warm climates and when the parrots cannot seek shade.

2. Environment
   - Thermal extremes are likely to fall beyond the parameters for effective thermoregulation, especially during transportation, but also if kept in a net or glue trap for some time
   - Close confinement with an absence of light and fresh air
   - Absence of enrichment/ barren enclosures
   - Unpredictable events/ noises likely when confined

Evidence for Environment welfare compromises

Grey parrots may be exposed to thermal extremes when confined in sacks, transported in small containers, or restrained in a glue trap or net, especially when in overcrowded containers, as is often seen [72]. The inability of the parrots to perform thermoregulatory behaviours can seriously impact their welfare and cause mortalities [266,267].

Confinement of birds is known to be detrimental to their welfare, especially for large-ranging birds like grey parrots [268]. This is further exacerbated by the fact that grey parrots are often traded in significantly overcrowded and small containers, with insufficient space to stretch their wings [72,74,265].

Unpredictable events and noises are known sources of stress for animals in captivity, especially when other senses, such as sight, are unavailable [269]. The parrots may be kept in these conditions for days or weeks.

3. Health
   - Risk of disease from close confinement and crowded housing
   - Risk of painful injuries from capture methods, including feather damage from glue traps and injuries from nets

- Young chicks are often unable to survive independently

Evidence for Health welfare compromises

Diseases are a common issue for trafficked grey parrots and are responsible for mortalities [72,75].

The capture methods used are known to cause considerable injuries to the parrots and can result in long-term pain [71,75,76].

Chicks who are too young to survive independently are often captured, resulting in them suffering slow deaths from starvation or thermal distress [79].

High mortality levels can result from trapping methods, and as many as 40% of parrots are thought to die before leaving the hunter, and a further 25% are estimated to die before they reach the market [79].

4. Behaviour
   - Barren and inappropriate environment, no freedom to make choices, and significant constraints on behaviour for long periods, including being unable to move, perch, stretch wings, feed, or drink
   - Negative interactions with humans

Evidence for Behaviour welfare compromises

Grey parrots experience considerable behavioural restrictions when captured, held, and transported. For example, parrots are commonly held and transported in overly crowded conditions, which prevent them from flying or even stretching their wings, and are not given access to perches [72].

As the parrots are wild and not habituated to humans, close contact, handling, and the pain of being removed from glue traps will result in negative human interactions [75,216].

5. Mental State: Potential affects arising from domains 1-4 include;
- ➢ (1) 1) Thirst and hunger
- ➢ (2) Discomfort, pain, stress, and fear
- ➢ (3) Sickness, pain, discomfort, fear, and stress
- ➢ (4) Exhaustion, frustration, fear, pain, and distress

Mental state welfare compromises

Welfare compromises in the previous four domains have the potential to give rise to a range of affects that African Grey parrots, as sentient beings, are known to be capable of experiencing [21].
