# Supplementary material for: Chains of Commerce: A Comprehensive Review of Animal Welfare Impacts in the International Wildlife Trade
Source: Animals (Basel). 2025 Mar 27;15(7):971. doi: 10.3390/ani15070971 (PMC11988014; doi:10.3390/ani15070971)
Supplement: Supplementary file 1 [file animals-15-00971-s001.zip › Table S4_Sharks.pdf]

Table S4: Sharks for the fin trade

Detailed explanation of the welfare compromises described in Table 2 for the trade of shark fins for traditional medicine.

Sharks for the fin trade
**Numbers:**<br>~63 – 273 million sharks per year (estimated calculation, although likely to be more when including illegal trade) [83].<br><br>Sharks are caught as bycatch and for their fins, meat, and oil in huge numbers yearly [83,87]. Worm et al. [83] conservatively estimated that around 97 million sharks were killed in 2010 alone, based on reported and unreported landings, discards, and shark finning data, and suggested that this could range from 63 to 273 million sharks per year. Although a significant proportion of these sharks are likely to be killed for their fins, determining the true scale of the shark fin trade is not possible, as importing shark fins is increasingly prohibited in countries worldwide [93, 96, 300]. Data retrieved from the FAO Global Fish Trade Statistics Database states that in 2020, 12,391 tonnes of shark fins were imported globally, indicating a decline from estimates reported in 2010, when over 17,000 tonnes were legally imported [84]. However, given the illegal nature of much of the trade in shark fins and the scale of unreported and regulated trade, these numbers are considered gross underestimations [85,86]. In fact, Bayesian models of trade data suggest that the true scale of the shark fin trade is around three or four times higher than the FAO estimates [85]. The main consumptive market for shark fins is in East and Southeast Asia, where shark fin soup is considered a delicacy. However, the trade in shark fins is a global issue, and the EU accounted for nearly half of reported total shark fin exports in 2020 [82, 301].
**Duration of experiences:**<br>Sharks are often returned to the sea alive following capture and fin removal. Some will be killed before fin removal.<br><br><u>Capture</u>: Hours to days<br>- Longline fishing or nets.<br><br><u>Fin removal</u>: Seconds to minutes<br><br><u>Slaughter</u>: Seconds to hours<br>- Most sharks will die from suffocation, exsanguination, or predation when thrown back alive.<br>- Landed sharks may be slaughtered by blunt trauma, but if not effective, then sharks will asphyxiate.

**Severity (welfare compromise using the Five Domains Model):**
1. <u>Nutrition</u><br>Restricted food intake (capture)
<u>Evidence for Nutrition welfare compromises</u><br>Sharks caught in a net or hooked on a long line will experience food deprivation for that time, typically ranging from minutes to hours, but could be longer [269]. The impact of feed deprivation for most species of sharks should be relatively low [302].
2. <u>Environment</u>
<u>Evidence for Environment welfare compromises</u><br>Since the sharks are kept in their natural environment for most of their experience during capture, and only brought on board for a short period, this domain is irrelevant.
3. <u>Health</u><br>- Injury from longline hook or net, and tools and landing procedure (capture)<br>- Severe injury and pain from fin removal<br>- Slow and inhumane death, suffocation, predation, exsanguination, or asphyxiation (slaughter)
<u>Evidence for Health welfare compromises</u><br>Sharks incur hooking injuries and netting injuries during the capture process, which can cause long-term pain and mortalities in escaped individuals [269]. The gaff tool used to hook and land sharks for finning also causes significant pain and injury, as the hook pierces the sharks' flesh [91].<br><br>The finning process causes considerable pain, as is typically performed on conscious individuals [91]. Sharks who are thrown back to sea alive then experience a slow and painful death from asphyxiation (for species that need to move for respiration), exsanguination, or predation from other fish, which they cannot escape from, all of which will cause extensive suffering rickets Sharks who are kept on board may be killed by blunt trauma, which can have limited effectiveness. Consequently, individuals may die from asphyxiation [93].
4. <u>Behaviour</u><br>- Severe restriction of behaviour when on longline, in a net, and post-fin cutting as cannot swim, escape predators, or feed (capture)<br>Negative interactions with humans (capture and fin removal)
<u>Evidence for Behaviour welfare compromises</u>

Sharks hooked onto a longline, entangled in a net, or post-finning, cannot perform normal behaviours, including escaping from predators.

Sharks are wild animals, so close contact with humans will likely be stressful. Furthermore, the landing and fin-cutting processes are extremely stressful and painful for the sharks, resulting in negative interactions with humans [91].

5. Mental State: Potential affects arising from domains 1-4 include;

- (1) Hunger
- (3) Pain, discomfort, fear, and stress
- (4) Exhaustion, frustration, fear, stress, pain, and distress

Mental state welfare compromises

Welfare compromises in the previous four domains have the potential to give rise to a range of affects that sharks, as sentient beings, are known to be capable of experiencing [18].
