# Supplementary material for: Chains of Commerce: A Comprehensive Review of Animal Welfare Impacts in the International Wildlife Trade
Source: Animals (Basel). 2025 Mar 27;15(7):971. doi: 10.3390/ani15070971 (PMC11988014; doi:10.3390/ani15070971)
Supplement: Supplementary file 1 [file animals-15-00971-s001.zip › Table S5_Pangolins_.pdf]

Table S5: Pangolins for traditional medicine

Detailed explanation of the welfare compromises described in Table 2 for the trade of pangolins for traditional medicine.

**Pangolins for traditional medicine**
**Numbers:** There are currently eight extant species of pangolins in Africa and Asia, and all of them are illegally traded internationally for their meat and scales [99–101]. Despite being listed in Appendix I of CITES in 2016, and banned from international commercial trade, they are still often cited as the most heavily trafficked CITES-regulated mammal [33,101–103].<br><br>Between August 2000 and July 2019, the equivalent of around 895,000 pangolins were estimated to be trafficked around the world, and in 2019, there were thought to be around 195,000 pangolins trafficked for their scales [99]. However, the actual figure will likely be much higher, as these figures are based on the portion of intercepted illegal trade [99]. One assessment of the exploitation of Central African pangolins (*Phataginus spp. a*nd *S. gigantea*) estimated that between 0.42 and 2.71 million pangolins were hunted each year in Central Africa between 1975-2014 [110]. Furthermore, this number is likely to be growing, as the Asian pangolins decline in number, and the currently more abundant African pangolins are used to fill the gap [99,109,110].
**Duration of experiences:**<br>Capture: Hours to days<br>- Opportunistic captures<br>- Hunting with dogs<br>- Dug out<br>- Smoked out<br>- Caught in trap/snare<br>- Speared<br>- Tightly tied in individual sacks<br><br>Transportation: Hours to weeks<br>- Typically transported in defensive balled position in net sacks, stacked upon one another<br>- Characterised by poor hygiene<br><br>Killing: Minutes to hours<br>- Hit or cut on the head

- Boiled for scale removal- some may still be alive

Severity (welfare compromise using the Five Domains Model):

1. Nutrition
   - Restricted food and water intake (capture and transportation)

Evidence for Nutrition welfare compromises

Once caught, pangolins are typically netted in their defensive balled positions and cannot unfurl [113]. This may last into the shipping process if traded alive. The pangolins are, therefore, unable to unfurl to feed or drink, and provisions are not typically provided [117].

2. Environment
   - Thermal extremes likely (capture and transportation)
   - Close confinement with an absence of light and fresh air (capture and transportation)
   - Unpredictable events/ noises are likely when held in a sack and carried around, as well as when transported (capture and transportation)
   - Barren environment (transportation)
   - Poor hygiene

Evidence for Environment welfare compromises

Once caught, pangolins are typically kept in their balled up position and cannot unfurl [113]. This means they may be exposed to thermal extremes and are prevented from performing natural thermoregulatory strategies to mitigate these.

Close confinement is stressful for animals, especially when bodily movement is so severely compromised [112]

Unpredictable events and noises are known sources of stress for animals in captivity, especially when other senses, such as sight, are unavailable [269].

3. Health
   - Dog bites (capture)
   - Smoke inhalation (capture)
   - Spear wounds/ infected injuries (capture and transportation)
   - Infectious diseases and parasites (capture and transportation)
   - Pain and stiffness from limited movement (capture and transportation)
   - Inhumane killing practices including hitting, cutting and boiling alive (killing)

- Crushing and suffocation (transportation)

Evidence for Health welfare compromises

Pangolins trying to escape may be caught by spear [106]. Dog bite wounds around the base of the tail are commonly seen in confiscated pangolins [117]. Confiscated pangolins are typically covered in faeces and urine and suffer from various infectious diseases and parasites [117]. Injuries, both from the hunting process and transportation, are also commonly seen in confiscated pangolins and individuals are often infected at the point of confiscation, which can result in loss of limbs or fatal septicaemia [117].

Many pangolins are 'processed' by the hunters themselves and are transported in the bag before being killed by being hit or cut on the head and then boiled for easier scale removal [106]. Some individuals may still be alive at the start of boiling, resulting in extensive pain and suffering [106].

- Behaviour
- Barren and inappropriate environment, no freedom to make choices, and significant constraints on behaviour for long periods, including being unable to unfurl (capture and transportation)
- Negative interactions with humans (potentially all phases)

Evidence for Behaviour welfare compromises

Pangolins are severely restricted once captured, as they are unable to uncurl their bodies, which prevents them from drinking, eating, or attempting to escape [117].

Pangolins are wild animals and are unlikely to gain any positive experience from interacting with humans. All interactions are considered to be highly negative (e.g., chasing, spearing, handling, killing).

4. Mental State: Potential affects arising from domains 1-4 include;

- (1) Hunger and thirst
- (2) Stress, frustration, pain, discomfort, fear, and exhaustion
- (3) Pain, discomfort, fear, sickness, and stress
- (4) Exhaustion, frustration, fear, stress, pain, and distress

Mental state welfare compromises

Welfare compromises in the previous four domains have the potential to give rise to a range of affects that pangolins, as sentient beings, are known to be capable of experiencing.
