# Supplementary material for: Chains of Commerce: A Comprehensive Review of Animal Welfare Impacts in the International Wildlife Trade
Source: Animals (Basel). 2025 Mar 27;15(7):971. doi: 10.3390/ani15070971 (PMC11988014; doi:10.3390/ani15070971)
Supplement: Supplementary file 1 [file animals-15-00971-s001.zip › Table S6_Crickets for feed and food.pdf]

Table S6: Crickets (*Gryllidae*) for feed and food

Detailed explanation of the welfare compromises described in Table 3 for the trade of crickets for food and feed.

Crickets for feed and food

Numbers:
~370 billion – 430 billion crickets are slaughtered or sold live or dead yearly [120].
34 billion – 41 billion crickets are alive on farms at any one time [120].

Insects are being heralded as the answer to the world's food security issues, as they can offer high protein sources and competitive conversion rates for both human food and livestock feed [119]. As a result, around the world, insect farms are increasing in size and number, but despite this, we still do not have a grasp of the welfare concerns involved [19,121,122]. In 2020, 370 billion – 430 billion crickets were thought to be slaughtered or sold live or dead per year, and an average of 34 billion – 41 billion crickets were estimated to be alive on farms at any one time [120]. Given the growing interest in farming insects, it is likely that this figure will continue to grow [118,120].

Duration of experiences:
Crickets are reared and slaughtered in a wide range of ways and systems.

<u>Rearing</u>: up to two months, longer for breeding individuals

<u>Slaughter</u>: seconds to hours

Severity (welfare compromise using the Five Domains Model):

1. <u>Nutrition</u>

- Food may be inappropriate, e.g. straw-based waste streams (rearing)
- Starved for a period before slaughter (slaughter)
- Water is usually available, although it may be limited to avoid drowning (rearing)

<u>Evidence for Nutrition welfare compromises</u>
Systems vary widely, and so does the type of food given to the crickets. But based on the worst-case scenario, as required by the Five Domains model [29], crickets may be fed inappropriate and limited feed sources, as done in some farms, such as straw-based waste streams. These fail to provide for the needs

of crickets and result in high levels of mortalities [126]. Most systems are thought to provide restricted feed sources and variety compared to what wild crickets would access [127].

Crickets are normally starved before slaughter, which introduces a welfare compromise depending on the duration [270,271]. Studies show that food restriction results in physical stress and poor growth in crickets, suggesting they do not cope with this welfare compromise [272]. Crickets will also resort to cannibalism if starved for too long.

2. Environment

- Overcrowding is a common concern (rearing)
- The environment is highly restricted in space and complexity compared with natural ranges (rearing)
- Air quality and temperature should be well controlled but can pose issues in less well-managed systems (rearing)

Evidence for Environment welfare compromises

Optimal conditions for rearing crickets are not always known or may be incompatible with what is required for high levels of production, a problem that is widespread across animal agriculture. In striving for the most efficient systems, insects may be given diets that are either unnatural or modified in a way that can impede their welfare.

Crickets will aggregate at certain times and in response to certain events, although these are temporary behaviours. Overcrowding may restrict the behaviours and ranges of the crickets, but it is unclear to what extent this affects them [122].

Crickets are highly susceptible to poor environmental conditions, including temperature and air quality changesand mortality levels will quickly rise [125].

In a poorly managed system, which is overcrowded and poorly managed in terms of environmental conditions, the welfare of the crickets is compromised, and mortality rates soon increase [125].

3. Health

- Risk of disease, injury, and cannibalism from overcrowding (rearing)
- Risk of mortality from misguided or poor husbandry practices (rearing)
- Potentially an inhumane slaughter method (slaughter)

Evidence for Health welfare compromises

Diseases are a significant concern, as an outbreak can kill a whole colony within hours [122].

Overcrowding and poor husbandry measures, including feed withdrawal or lack of sufficient nutrients in the feed, can result in crickets becoming cannibalistic [270,271].<br><br>Crickets and other farmed insects are, however, less able to cope with inadequate conditions than vertebrate livestock and suffer inordinately high mortality rates [125]. This is partly due to the lack of veterinary knowledge of insects, as well as a dearth of knowledge of the pests and diseases that are most problematic [273].<br><br>As the full breadth of the needs of crickets is still not understood, there is a potential for misguided husbandry protocols to cause unnecessary suffering and welfare issues [273]. Similarly, knowledge in dealing with and recognising, diseases in crickets is lacking, which may result in unnecessary suffering and mortalities [273].<br><br>Most killing methods for crickets are based on the integrity of the end product rather than any consideration for the insect's experience [273]. For example, methods may be chosen based on a need to deal with microbiological load [274], or to maintain the sensory and physiological properties of the insect product [275]. Methods to kill crickets include heating, shredding, and freezing [273]. Some farmers attempt to render them unconscious first by gassing them with carbon dioxide or by chilling them before freezing them [270,276]. However, there is very little evidence guiding best practices in this area.
4. <u>Behaviour</u><br>Barren and restricted environment, with no freedom to make choices and significant constraints on behaviour for days, potentially weeks. Although unclear yet what impact this may have on crickets (rearing).
<u>Evidence for Behaviour welfare compromises</u><br>The behaviour of farmed crickets is severely restricted, as they are kept in far smaller areas than they would normally inhabit. Furthermore, their captive environments tend to be barren and lacking in complexity, compared with the wild, and prevent natural social dynamics [19,277]. It is unclear, however, to what extent this impacts the welfare of crickets, although based on the worst-case scenario, these welfare impacts could cause considerable frustration and stress in the crickets.
5. <u>Mental State</u>: Potential affects arising from domains 1-4 include;<br>➢ (1) Hunger<br>➢ (2) Discomfort, pain, stress, and fear<br>➢ (3) Sickness, pain, discomfort, fear, and stress<br>➢ (4) Frustration and stress

Mental state welfare compromises

Welfare compromises in the previous four domains have the potential to give rise to a range of affects that crickets may be capable of experiencing [19,124,278].

The extent to which the crickets' welfare is compromised by these impacts is unknown, and so the welfare concerns described in the previous four domains represent not only the worst-case scenario in terms of the farm's practices but also in terms of the extent of suffering a cricket would experience as a result. Similarly, for the fifth domain, the allocated affects are based on the assumption that crickets can suffer welfare compromises, although the extent to which they impact their mental states is still unclear.
