# Supplementary material for: Chains of Commerce: A Comprehensive Review of Animal Welfare Impacts in the International Wildlife Trade
Source: Animals (Basel). 2025 Mar 27;15(7):971. doi: 10.3390/ani15070971 (PMC11988014; doi:10.3390/ani15070971)
Supplement: Supplementary file 1 [file animals-15-00971-s001.zip › Table S7_frogs.pdf]

Table S7: Frogs (*Anura*) for the frog-leg trade

Detailed explanation of the welfare compromises described in Table 3 for the trade of frogs for the frog-leg trade.

**Frogs for the frog-leg trade**
**Numbers:**<br>~100-400 million per year internationally [138].<br>~814 million to 2 billion frogs were imported into the EU alone between 2011-2020 [134].<br><br>The frog-leg trade is a sizable industry, and the EU alone imported around 40,7000 tonnes of frogs' legs between 2011-2020, equating to between 814 million and 2 billion individual frogs [134]. Most went to Belgium (70%), but France (16.7%) and the Netherlands (6.4%) also imported considerable amounts [134]. Frog legs are also consumed in North and South America, Africa, and Asia [137,279,280]. Indonesia is the main supplier of frogs' legs, followed by Vietnam and other Asian countries, and although farming of frogs for the trade is on the increase due to depleted wild populations, in part caused by over-exploitation, the majority traded are still sourced from the wild, incorporating a wide diversity of species [134,136,279].
**Duration of experiences:**<br>Frogs are captured and then transported to 'holding facilities', buyers, or directly to markets. Frogs are typically slaughtered within hours or days of capture, although some may be transported live for consumption.<br><br><u>Capture and market</u>: Hours to days<br>- Caught by hand, spear, or net<br>- Held in overly crowded buckets piled on top of one another<br><br><u>Slaughter</u>: Seconds to hours<br>Legs are cut off with scissors or a knife or pulled off by hand—no pain relief or stunning.
**Severity (welfare compromise using the Five Domains Model):**
1. <u>Nutrition</u><br>- Restricted water availability and food intake (capture and market)
<u>Evidence for Nutrition welfare compromises</u>

Different frog species have different tolerances to feed and water deprivation, and as frogs consume water through their skin, their tolerance also depends on their environmental conditions [281,282]. Frogs show adaptive behaviour regarding feed availability, and some species can withstand long periods of days or weeks without food [8].

The needs of different frog species vary, though, and their tolerance is also impacted by environmental conditions, resulting in different welfare experiences.

2. <u>Environment</u>

- Thermal extremes are likely, and potentially, an absence of damp substrate or water (capture and market)
- Close confinement, often with an absence of light, fresh air, and water, and potentially in a pile of other animals (capture and market)
- Unpredictable events/ noises are likely when held in a bucket with other animals and carried around (capture and market)

<u>Evidence for Environment welfare compromises</u>

Frogs consume the majority of their water needs through their skin. Therefore, the availability of water for them to soak in and the optimum humidity levels are important for their welfare and can be significantly compromised during trade, potentially resulting in mortality, as they are often kept in barren and over-crowded containers [281, 303).

Frogs collected for trade are often placed in a bucket filled with other frogs, grossly limiting the space available, the amount of air, and the water available to them, which can often result in mortalities [134,283].

Unpredictable events and noises are known sources of stress for animals in captivity, especially when other senses, such as sight, are unavailable [248].

The frogs may be kept in these conditions for days or weeks at a time.

3. <u>Health</u>

- Risk of disease and injury from close confinement (capture and market)
- Risk of suffocation and crushing from holding method (capture and market)
- Risk of injury and mortality from capture method (capture and market)
- Inhumane, slow, and painful slaughter (slaughter)

<u>Evidence for Health welfare compromises</u>

Disease is a common issue for frogs traded for frog legs, and this has been known to facilitate the spread of deadly pathogens, which affect not only those traded frogs but can also impact and threaten other wild frogs [138,284].

As frogs are piled on top of one another, suffocation and crushing are a risk when they are caught and transported in an over-crowded container [134,138,283]. Injuries and mortalities during capture and subsequent transportation are common issues, especially when roughly handled or spears are used for capture [137]. As a result, exporters often refuse frogs as they are too badly bruised and damaged [137].<br><br>The slaughter methods used, including spears during capture or the removal of their legs via scissors, knives, or hand dismemberment in live frogs, cause significant pain and suffering to these conscious animals [134,136]. Furthermore, the frogs may not die immediately from this method, resulting in a slow and painful death [134,136].
4. Behaviour<br>- Barren and inappropriate environment, with no freedom to make choices and significant constraints on behaviour for days, potentially even weeks (capture and market)<br>- Negative interactions with humans (all phases)
Evidence for Behaviour welfare compromises<br>The behaviour of the frogs during this stage is grossly compromised by overcrowding and significant confinement, potentially for weeks at a time. Furthermore, the frogs cannot engage in important natural behaviours such as feeding, locomotion, resting, and accessing water or damp areas.<br><br>The frogs' experience and interactions with humans are negative, not only because frogs find handling to be stressful [17], but also because of the severe pain from rough handling and slaughter methods inflicted upon them [134,136].
5. Mental State: Potential affects arising from domains 1-4 include;<br>➢ (1) Dehydration and hunger<br>➢ (2) Discomfort, pain, stress, and fear<br>➢ (3) Sickness, pain, discomfort, fear, and stress<br>➢ (4) Exhaustion, fear, and distress
Mental state welfare compromises<br>Welfare compromises in the previous four domains have the potential to give rise to a range of affects that frogs, as sentient beings, are known to be capable of experiencing [283].
