# Supplementary material for: Chains of Commerce: A Comprehensive Review of Animal Welfare Impacts in the International Wildlife Trade
Source: Animals (Basel). 2025 Mar 27;15(7):971. doi: 10.3390/ani15070971 (PMC11988014; doi:10.3390/ani15070971)
Supplement: Supplementary file 1 [file animals-15-00971-s001.zip › Table S8_crocs.pdf]

Table S8: Crocodilians (Crocodylidae) farmed and killed for their skins

Detailed explanation of the welfare compromises described in Table 4 for the farming of crocodilians for their skins.

**Crocodilians farmed and killed for their skins**
**Numbers:**<br>Numbers vary on farms, but there are thought to be around 5000 crocodilian farms worldwide [142].
**Duration of experiences:**<br>Rearing:<br>Crocodilians are typically captive-bred or ranched after harvesting eggs from the wild. Rearing occurs from hatching until they reach slaughter weight at around 2-3 years old, depending on the species [2].<br><br>Killing:<br>Investigations and studies suggest that killing methods may result in the animals being conscious when processed, and so killing may take minutes to hours, depending on the processes and time taken.
**Severity (welfare compromise using the Five Domains Model):**
1. Nutrition<br>- Insufficient information
2. Environment<br>- Overcrowding<br>- Severe confinement<br>- Barren conditions<br>- Unnatural environment
Evidence for Environment welfare compromises<br>For the first phases, crocodilians are typically kept in concrete-floored pens that are far removed from their wild habitats [160]. They are also kept in unnatural overcrowded groups despite being largely solitary.<br>In the last phase of rearing, crocodiles reared for their belly skins, such as Saltwater crocodiles, are kept in very restricted solitary pens with very little space to move [2].
3. Health

- Numerous diseases<br>- High levels of captivity stress<br>- High mortality and premature deaths<br>- Inhumane, slow and painful deaths
<u>Evidence for Health welfare compromises</u><br>Crocodiles are sensitive to stress, and intensive, unhygienic rearing conditions can trigger disease outbreaks (e.g., Crocodile pox, Caiman pox, Chlamydiosis, Dermatophilosis and Salmonellosis [156].<br>West Nile virus (WNV) reportedly affects various farmed crocodylian species (e.g., *Alligator mississippiensis, Crocodylus niloticus, Crocodylus moreletii*; [285] which can cause premature death (mortality rates of 40-60% have been reported; [285]) and a variety of neurological manifestations, such as swimming in circles, ataxia, and head and muscle tremors [285]. Since 2001, there have been multiple reported outbreaks of WNV in farmed American alligators (*Alligator mississippiensis*) in the US [285,286].<br>Mortality can also occur due to an inability to thermoregulate, exposure to fluctuating temperatures, or overheating [156].<br>Average hatchling mortality rates are 13.4%, although deaths due to disease and known stressors (e.g., grading and moving) are reasonably low (1.6% and 0.98%) [287].<br><br>Despite claims that killing is performed humanely, investigations and studies have found that methods are frequently inhumane and result in individuals still being conscious and sensible to pain whilst being processed and skinned alive [157,158,164,288].
4. <u>Behaviour</u><br>- Severely restricted<br>- Unnatural overcrowding/ social grouping
<u>Evidence for Behaviour welfare compromises</u><br>For the first phases, crocodilians are kept in unnaturally crowded groups. They are an aggressive species and can develop co-occupant aggression in close proximity to others [155,287]. The barren pens they are in thwart their natural behaviours and instincts and severely restrict movement. The pens they are kept in towards the end of their captive lives significantly restrict movement to protect the belly leather [6].
5. <u>Mental State</u>: Potential affects arising from domains 1-4 include;<br>➢ (2) Discomfort, pain, stress, frustration, and fear<br>➢ (3) Sickness, pain, discomfort, fear, frustration, and stress<br>➢ (4) Exhaustion, fear, frustration, anxiety, pain, and distress
<u>Mental state welfare compromises</u>

Welfare compromises in the previous three domains have the potential to give rise to a range of affects that crocodilians, as sentient beings, are known to be capable of experiencing [20].
