# Supplementary material for: Chains of Commerce: A Comprehensive Review of Animal Welfare Impacts in the International Wildlife Trade
Source: Animals (Basel). 2025 Mar 27;15(7):971. doi: 10.3390/ani15070971 (PMC11988014; doi:10.3390/ani15070971)
Supplement: Supplementary file 1 [file animals-15-00971-s001.zip › Table S9_Lions.pdf]

**Table S9: Lions (*Panthera leo*) on commercial farms**

Detailed explanation of the welfare compromises described in Table 5 for the farming of lions for entertainment.

| **Lions on commercial farms** |
|---|
| **Numbers:** Commercial lion farming is a significant and growing industry in South Africa, with around 8500 lions estimated to be registered at nearly 400 facilities across the country, contributing around US$42 million annually to the South African economy [165,166]. |
| **Duration of experiences:**<br>Farming: (Years)<br>- Kept on farms as cubs for tourist petting experiences and then as adolescents and adults for 'lion walking' experiences<br><br>Killing: (Seconds to days)<br>- Canned hunting<br>- Killed for body parts (for traditional medicine)<br>- Typically shot, but the duration is dependent on the effectiveness of the shot (canned)<br>- May die from infections or injuries |
| **Severity (welfare compromise using the Five Domains Model):** |
| 1. Nutrition<br>- Cubs and adults are often found to be malnourished. Insufficient and poor-quality food provided.<br>- Cubs are typically weaned too early, and appropriate milk replacer is not always provided.<br>- Water is often unhygienic or not provided freely. |
| Evidence for Nutrition welfare compromises<br>Surveys of lion farms have found numerous cases of malnourished lions and cubs [170]. Cubs are weaned too early, making them reliant on alternative food sources, which are notoriously difficult to replicate for young cubs, as milk formulas do not typically have the right nutritional elements required by lions (e.g. taurine) [165]. |
| 2. Environment<br>- Severe confinement and inappropriate housing for cubs and adults. |

- Barren housing, lack of shelters, and no environmental enrichment.
- Lions are often made to walk in severe heat.

Evidence for Environment welfare compromises

Surveys have found lions on commercial lion farms are typically kept in severely confined enclosures, often subject to overcrowding [170].

Environmental enrichment and shelters also tend to be missing [170]. A lack of environmental complexity and appropriate space are known to be significant factors for the welfare of big cats, especially as they occupy large ranges in the wild [289].

Barren environments are also significant causes of stress for captive animals [290].

Overcrowding and inappropriate housing can result in increased aggression in the lions, which can also impact the other domains [170].

Lionesses are not typically given appropriate housing for birthing, nursing, and rearing cubs, which may result in chronic stress [171].

Lions typically rest in the heat of the day and may be repeatedly walked or interacted with during all times of the day. This can impact their biological rhythms, disrupt their natural behaviour, and result in thermal stress [165].

3. Health
   - High prevalence of injuries from training methods, aggressive encounters with other lions, and rough handling.
   - Poor health results from malnutrition.
   - Risk of fractures from bone weaknesses.
   - Thermal stress from exposure to high temperatures.
   - Physical exhaustion from being overworked.
   - High prevalence of diseases and early mortality.
   - Pain from inhumane killing methods.

Evidence for Health welfare compromises

Surveys show that lions on these commercial farms suffer from numerous health issues, including injuries from aggressive interactions, as the result of poor management [170]. Furthermore, these injuries are often not properly treated due to a lack of specialised veterinary care [170].

Rough handling may also result in fractures and handling stress, especially as these wild animals are not only unaccustomed to human handling, but they are also susceptible to weak bones and poor immunity from malnutrition [170].

Naturally, lions will rest in high temperatures as a form of behavioural thermoregulation. Exposure to high temperatures may result in thermal stress, and exhaustion and is at odds with their natural behavioural instincts [170].

Diseases are commonly seen in commercial lion farms and can result in early mortalities [170]. As veterinary care is often limited, this can result in unnecessary suffering for the lions, which may be long-term.

There is little information on the killing methods used for lions who are killed for the Traditional Medicine trade. Given the evidence from other species in this trade, it is highly probable that methods will not necessarily be performed with the animal's wellbeing in mind and may result in unnecessary pain and suffering.
Furthermore, some lions may die from untreated injuries and disease or be deliberately neglected until death.

Lions used for canned hunting experiences are subject to being killed by potentially novice hunters. These hunters may not effectively kill the lion on the first hit, and subsequently, the lion may be left to suffer for hours or even days before death [168].

4. Behaviour

- Barren and restricted environment, no freedom to make choices, and significant constraints on behaviour for entire life.
- Early removal from the mother results in maladapted individuals and stressed mothers.
- Unable to retreat or refuse interactions with humans.
- Abnormal social groupings and continual separation from peers result in stress, fear, and aggression.
- Forced and negative interactions with humans both during training and with tourists.
- Unnatural behavioural patterns due to overworking.

Evidence for Behaviour welfare compromises

Barren and restricted environments have significant impacts on animal welfare and behaviour and can result in long-term stress and suffering [291].

Early weaning and removal from the mother negatively impact both the cubs' and the lionesses' behaviour, as they cannot perform natural behaviours and experience considerable stress as a result [171]. Furthermore, this also results in lionesses rejecting future litters, as the repeated early removal of cubs causes them chronic stress, which manifests through poor maternal behaviours [171].

Lions cannot reject interactions with humans and may be forcibly trained to interact, often using inhumane methods [165,171]. This lack of control can have significant and long-term negative impacts on their welfare and will likely result in stressed and fearful animals [165,171].

| |
|---|
| Lions typically rest for long periods, especially young cubs, and impeding these behaviours can lead to exhaustion, physical and psychological stress, and frustration in these animals, especially as they cannot exert control [165,171]. |
| 5. <u>Mental State</u>: Potential affects arising from domains 1-4 include;<br>➢ (1) Hunger and thirst<br>➢ (2) Discomfort, pain, stress, and fear<br>➢ (3) Sickness, pain, discomfort, fear, frustration, and stress<br>➢ (4) Exhaustion, fear, frustration, pain, and distress |
| <u>Mental state welfare compromises</u><br>Welfare compromises in the previous four domains have the potential to give rise to a range of affects that lions, as sentient beings, are known to be capable of experiencing [21]. |
